# Artificial Neural Networks for Predicting Emissions from the Livestock Sector: A Review

**DOI:** 10.3390/ani16010101

**Published:** 2025-12-29

**Authors:** Luciano Manuel Santoro, Provvidenza Rita D’Urso, Claudia Arcidiacono, Giovanni Cascone, Salvatore Coco

**Affiliations:** 1 Building and Land Engineering Section, Department of Agriculture, Food and Environment (Di3A), University of Catania, via S. Sofia 100, 95123 Catania, Italy; luciano.santoro@phd.unict.it (L.M.S.); carcidi@unict.it (C.A.); gcascone@unict.it (G.C.); 2 Department of Electrical, Electronics and Informatics Engineering (DIEEI)—Section of Electrical, Electronics and Systems Engineering, University of Catania, via Santa Sofia n° 64, 95123 Catania, Italy; salvatore.coco@dieei.unict.it

**Keywords:** artificial neural network, livestock production, emission, machine learning, housing system, manure management

## Abstract

Gaseous emissions from livestock facilities pose environmental and health concerns. Monitoring pollutant gases is essential to mitigate impact and enhance the sustainability of livestock systems. Emerging Artificial Intelligence (AI) technologies—particularly Artificial Neural Networks (ANNs)—offer advanced tools to address these challenges by improving livestock monitoring and management. Following PRISMA guidelines, 18 studies published between 2007 and 2024 were selected from Web of Science^®^ and Scopus^®^. Most research was conducted in Europe (55%), primarily focusing on cattle and swine. Among gases, ammonia (NH_3_) was predicted in 50% of studies and methane (CH_4_) in 35%. The most common ANN architecture was the Multilayer Perceptron (MLP), trained mainly with backpropagation algorithms and validated using the Root Mean Square Error (RMSE). The results show that ANN models consistently outperformed traditional statistical approaches, offering greater prediction accuracy. Future research should focus on identifying optimal ANN structures for precise emission prediction, accounting for environmental variability, reducing dataset bias, and combining ANN with statistical models to develop hybrid approaches that further improve livestock management and sustainability.

## Simple Summary

Artificial Intelligence (AI) is increasingly becoming an important tool for problem-solving in livestock farming, particularly in relation to sustainability. This study reviews the scientific literature to examine where these smart applications are being developed and how they are being applied. The analysis reveals that most research is currently taking place in Europe, with a primary focus on predicting environmental issues, such as harmful gas emissions from farms. Among the available technologies, the most common are data-driven computer models that emulate human learning processes and are validated against real-world data. In conclusion, this research demonstrates that AI-based approaches can generate accurate predictions to better guide farm management practices, supporting farmers and policymakers in decision-making processes that enhance the efficiency, economic viability, and environmental sustainability of livestock systems.

## 1. Introduction

The increasing demand for animal products, due to the expanding of the global population, defines a new challenge for livestock farming and related industries [1,2]. This challenge is to improve the balance between optimising productivity and ensuring environmental sustainability [3], as well as scale-up production and address the interaction between food security and environmental management [4]. In detail, the global livestock population will be over 100 billion, demanding a third of the world’s fresh water, and global feed is expected to be doubled as the global population continues to increase [5]. Negative effects on the environment (i.e., eutrophication, soil acidification, global warming, air pollution, increase in water consumption) are caused by different livestock sources: emissions [6], soil, feed and water consumption [7], and energy usage from fossil sources [8].

In the literature, livestock pollution emissions are a growing concern among researchers [9]. In particular, livestock emissions are well-known as being affected by housing system [10], waste management [11], ventilation systems [12], animal diet and nutrient loss [13], as well as food production [14]. The concentrations of pollutants are key factors in ensuring robust estimation methods, as they increase accuracy due to their strong correlation with emission levels [15,16,17].

Therefore, the challenge is a paradigm shift towards practices that ensure long-term ecological resilience, biodiversity preservation, and mitigation of the environmental impacts in livestock production. Within this context, the introduction of innovative strategies, such as Precision Livestock Farming (PLF), will be imperative to reduce the emissions from livestock production [18]. PLF strategies are based on real-time monitoring of farms and barns, thanks to the application of devices able to make decisions and collect data [19,20].

Emerging technologies using Artificial Intelligence (AI) have provided livestock farming with advanced solutions to complex problems, addressing livestock monitoring and management needs to optimisation and cost reduction [21]. Born in late 1940, AI is defined as an approach able to solve complex problems in such a way that can resemble humans. Applications of this tool are focused on evaluating dataset, autonomous decision-making, and surveillance and detection [22].

Machine Learning (ML) is a subset but evolved AI application where advanced algorithms and models can learn from data without any specific programming, improving the performance of the task assigned and identifying and analysing complex linear and nonlinear patterns [23]. The application of ML approaches has been one of the innovative strategies to analyse and assess livestock production processes and animal-related activities [24].

Classical statistical approaches, such as linear regression (LR), partial least square (PLS), autoregressive integrated moving average (ARIMA), analysis of variance (ANOVA), ridge regression (RR), multivariate linear regression (MLR), and polynomial regression (PL), are based on models structured on logical variables (risk factors) and different algorithms [25]. For instance, in D’Urso et al. [26], dependence on gas distribution in a dairy barn on those factors was assessed by applying one-way ANOVA and Tukey’s tests. In Rodrigues et al. [27], the authors used the Kruskal–Wallis test to compare emissions during different seasons and years of measurement, and Dunn’s post hoc test was applied for the respective multiple comparison in those studies; however, no predictions of gaseous emissions were carried out. Hempel et al. [28] analysed and modelled emissions by using a multi-linear regression model where climatic variables were independent parameters.

Compared to classical approaches [29,30], ML has shown better performance and accuracy [25,31]. In the literature, ML approaches have been applied in several studies [32,33,34]. ML consists of algorithms and statistical models—random forest (RF), decision tree (DT), partial least square (PLS), gradient boost decision tree (XGBoost), and support vector machines (SVM)—that computer systems use to learn and perform a specific task without being explicitly programmed [35,36,37].

In recent years, advanced ML models and techniques such as artificial neural networks (ANN) have spread in the scientific literature due to their high efficiency and effective processing approach to handle datasets and derive information [38]. The structures emulate the human brain with algorithms and connections adapting to the tasks given and learning by performing. Each ANN is structured in layers (i.e., input, hidden, and output), connected with neurons. Input layers receive input data and select variables; hidden layers process variables through activation functions and weights, understanding pattern and connection with variables and outputs; output layers make predictions [39]. Before ANN’s training starts, dataset is grouped randomly in three separated sub datasets (i.e., training, validation, and test). Training data trains model according to weights and biases; validation data tune hyperparameters selected (i.e., algorithms, learning rate, and number of neurons), avoiding overfitting; test data evaluate the final model performance [40]. Each time ANN completes a cycle of iterations, update its weights and parameters, according to the training occurred. Cycles are called epochs, and their number can usually range from 100 to 1000. A higher number of epochs corresponds to higher accuracy of the ANN models [41].

Their application in livestock systems have improved knowledge in different fields: lactation milk yield and field quality [42], genetic prowess and meat quality [43,44], sorting management [45], and health monitoring [46,47,48]. The use of ANNs have supported and improved animal science due to the versatility and potential of ANN methodologies that apply big data analysis, advanced models, and algorithms [49].

In the field of agriculture-related environmental issues, some international estimation guidelines, such as those from the Intergovernmental Panel on Climate Change (IPCC), or international projects, for example, DATAMAN [50,51], have been applied to evaluate the emissions of greenhouse gas (GHG), such as methane (CH_4_) and nitrous oxide (N_2_O) [52,53], and ammonia (NH_3_) [54,55]. Since these approaches have some limitations due to the data quality, consistency, availability, and accessibility [56], a further step to increase their accuracy and performance was carried out by integrating ANN models in them with the opportunity of managing and processing big data in real-time. In addition, ANN can make decisions and obtain information from different kinds of sources like images and environment. An approach of this kind was proposed by Kiat et al. [57], who suggest using ANN models to evaluate CH_4_ impact in climate change with the application of IPCC guidelines to assess emissions. As has been performed by Kolasa-Więcek [58], the authors applied ML and ANN tools in PLF to assess decision-making models. However, in these studies, there is a lack of any information about the ANN structures tested, including associated training algorithms, hyperparameters, hidden layers, and datasets.

As sophisticated ML models, ANNs can navigate and process large datasets and enhance the accuracy of livestock productivity forecasts thanks to tuned hyperparameters and specific ANN structures [59]. Currently, ANNs are promising in analysing complex systems, such as livestock farming, where the complexity of the data collected (i.e., big volumes of data, diverse range, and heterogeneity of physical variables) requires advanced approaches.

Since ANNs are applied to learn from data, make decisions, and adapt to extensive and complex datasets, they can be used in understanding and predicting emissions from livestock productions [60]. Recognising the potential of ANNs in this context, this review provides detailed analyses of the ANN models, disclosing their main features, structures, parameters and hyperparameters, and how the use of ANNs could be applied to evaluate emissions impacts from the livestock sector, supporting new or early researchers, and disclosing the gaps and limitations of the current state of the art. In detail, this review starts from the analysis of the existing literature to identify specific research needs and, consequently, formulate research questions (see Section 2.1).

The novelty of this review derives from the new perspective on the evaluation of the state of the art, which investigates the specific ANN characteristics and model applications to livestock gaseous concentrations and emissions, providing a detailed and specific framework for researchers interested in ANN applications, highlighting the gaps of the ANN models applications and making specific proposals for further research. In detail, the analysis has been carried out with specific attention to the livestock context analysing the sustainability of production, the livestock farming management, the farming monitoring systems in a wide perspective to understand patterns and interdependencies when applying ANN models.

## 2. Aims and Methodology

### 2.1. Research Needs and Research Questions

The first step in defining the research questions was to analyse the existing reviews in the field, where the objectives were identified, and the contribution to the advancement of research was highlighted. In detail, five reviews, showed in Table 1, investigated the application of ML and ANN for analyses, predictions, and evaluations of gaseous emissions from livestock farming [42,43,46,49,56]. In Table 1, the significative information derived from the five reviews are grouped into fields related to the number of articles and period analysed, aim of the review, methodology applied, livestock and gases considered, equipment for data gathering, ML and ANN models applied, and main results of the reviews.

In detail, Niloofar et al. [56] reviewed and categorised ML and ANN approaches in livestock farming, showing their potential in several fields of application in PLF management. Although IPCC methodology lacks optimisation approaches to estimate GHG emission, the tested ML and ANN models were able to accurately estimate emissions. However, authors statements lack any information of layers and neurons applied in the reviewed articles.

In Shine and Murphy [42], the authors carried out a literature review to map the recent trends in ML applications in dairy farms. They mapped the publications in a timeframe from 1999 to 2021 and underlined geographical distribution around the world. In addition, they clustered the publications regarding the aim and type of the ML (i.e., regression, tree analysis, classification, clustering, bayes, and ANN), the field of research (i.e., health, husbandry, behaviour, management, feeding, milk), and the validation criteria (i.e., correlation coefficient (r), mean absolute error (MAE), root mean square error (RMSE), mean square error (MSE), concordance correlation coefficient (CCC), coefficient of determination (R^2^) and mean absolute percentage error (MAPE), relative prediction error (RPE), mean squared percentage error (MSPE), and mean percentage error (MPE)). However, only two reviewed studies have considered GHG emissions without providing any specific information on the gas analysed.

Researchers evaluated several ANN applications in animal breeding, in vitro prediction of CH_4_ and CO_2_, forecast of milk yield and genome, evaluation of meat, inference demography and recombination, animal nutrition, and animal reproduction management [43]. In this review, however, any specific characteristics regarding ANN structures is missing, although it contributes to the knowledge in the field by reporting on different ANN models applied in livestock management productions. In a similar way, [46] assessed the application of ML and ANN models in infrared spectrometry applied in livestock systems to predict complex phenotypes. The models have been used in prediction approaches like milk composition, feed intake, energy balance, CH_4_ emission, fertility, health status, and meat traits. As in Rahman et al. [43], the authors did not specify any ANN model structures used in the articles they reviewed. In Jiang et al. [49], the authors carried out a review regarding the impact of energy cost in CH_4_ emissions and agricultural productions from data collected between 1981 and 2012. The authors applied two different methods, Box–Jenkins and undefined ANN models, to assess their prediction. However, there are no predictions neither on other GHGs nor on NH_3_ concentrations and emissions.

The analysis of the reviews highlights that there is a lack in the literature on the application of ANN models to sustainability of livestock housing, especially regarding models to assess gas concentrations and/or the emissions from livestock farms. In addition, evaluations of the different ANN training algorithms and structures are missing in the recent literature reviewed. Therefore, this review contributes to filling those research gaps in the livestock sector by providing a defined framework that is in line with the Preferred Reporting Items for Systematic Reviews and Meta-Analyses (PRISMA) model and includes graphical elaboration to support the findings of this review. Under this perspective, this review explores NH_3_ and GHGs concentrations and emissions by using ANNs. From the publications analysed, the following main research question have been formulated:

How are artificial neural network facing the challenges and innovating in gas concentration and emissions prediction in livestock farming?

In addition, specific research questions (RQ) were identified to assist in the presentation of the results:

RQ1. What is the regional distribution of the bibliographic research?
RQ2. What are the ANN applications and devices applied in gas estimation?
RQ3. What types of species and livestock management are the most investigated?
RQ4. Which ANN structures are prevalent and minor in current approaches?
RQ5. What are the characteristics (i.e., pre-processing, timeframe, parameters, temporal evolution) of the datasets that are predominantly used?
RQ6. What type of evaluation metrics have been applied?
RQ7. Which ANN training algorithms have been used?
RQ8. Which comparisons have been made among statistical, ML, and ANN approaches in the reviewed articles?

In Table 1, the contribution of the reviews to the identified RQ was added in the Results column, with the aim of considering the basic knowledge from the previous literature reviews.

### 2.2. Review Development Methodology

The systematic literature review was conducted following the PRISMA framework. To perform this analysis, a literature search was carried out on the Web of Science^®^ and Scopus^®^ databases, focusing on the studies published in the most recent years (i.e., 2007–2024). The following keywords were utilised for the search: “neural” AND “network” AND “livestock” AND “emission” AND “concentration”. Exclusion and inclusion criteria were applied to guide the optimal development of the review, avoiding redundancy and off-topic papers. The results exclude articles not written in English and outside the timeframe mentioned above. In addition, only articles were considered for inclusion, excluding conference papers, technical communications, and review articles. Duplicate studies were also identified and removed from the analysis.

**Figure 1:**
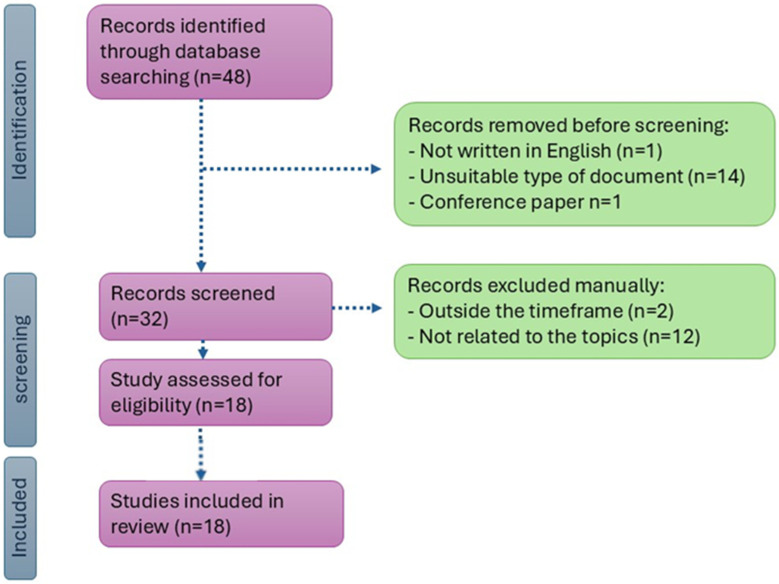
Flow chart of scientific studies from identification to inclusion stage, in line with inclusion and exclusion criteria.

The selected keywords were searched into title keywords and abstracts of the databases, providing the selection of 48 publications. From this selection, the application of exclusion criteria limited the number of publications, excluding one non-English publication, two studies outside the designated time frame, twelve publications with study’s objectives not in line with this research study, and fourteen identified as conference papers (eight), reviews (five), or a book chapter (one). Based on this selection, the data were systematically extracted from the remaining 18 publications (Figure 1). Then, these studies were analysed acquiring information to answer the eight RQs.

VOSviewer 1.6.20 software was applied to realise a visual network landscape. The publications’ references were downloaded as a .ris file from the Scopus and WoS databases, while correlated keywords were included from titles and abstracts. Full counting was applied to the network, and a minimum of four occurrences were chosen to meet the threshold; 53 keywords were selected by the software. Ten of these terms were evaluated as being eligible for the visualisation of the landscape.

## 3. Results

### 3.1. Articles Geographical Distribution and Bibliographic Cluster Analysis (RQ1)

**Figure 2:**
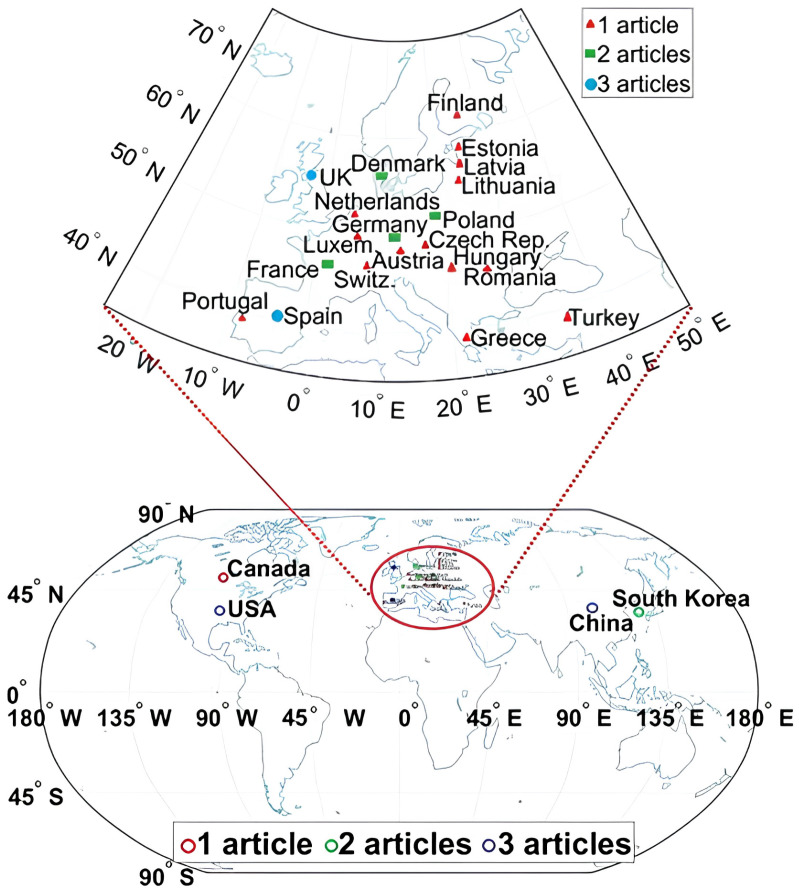
Geographical distribution of the papers selected.

Resulting from the PRISMA-based analysis of the publications found in the literature, Table 2 provides an overview of the data acquired from the selected research studies. The eighteen studies analysed data acquired in 27 countries with the following distribution: three from the United States, Republic of KoreaRepublic of Korea, Spain, and China; two from Germany, Poland, France, the United Kingdom, and Denmark; and one from Switzerland, Bulgaria, the Czech Republic, Canada, Estonia, Greece, Latvia, Lithuania, Luxembourg, Hungary, the Netherlands, Austria, Portugal, Romania, Slovenia, Slovakia, and Finland (Figure 2). The results show that Europe is the main contributor with 55% of the research studies analysed, Asia represents 39% of the dataset after Europe, and the remaining continents together contribute for the 22%. Additionally, the global distribution of the identified studies is illustrated in Figure 2.

**Figure 3:**
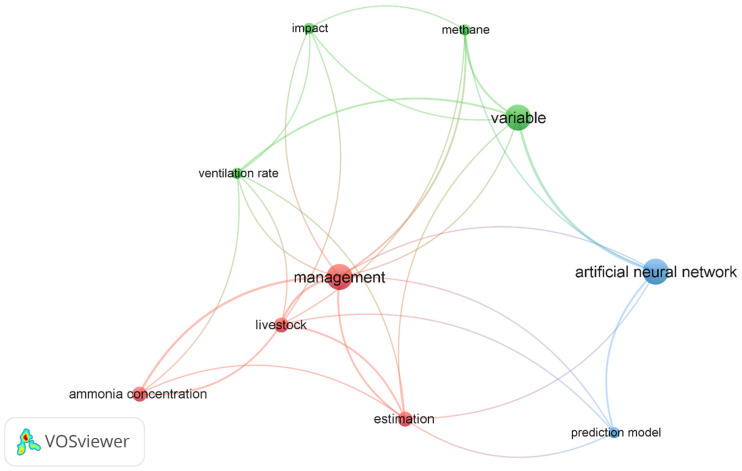
Visualisation network map for the papers selected. The three colours indicate the main clusters while the sizes of each nodes represent the occurrence value.

A VOSviewer network data map has been created to present the citation occurrence between the papers selected (Figure 3). Ten keywords (including titles and abstracts), binary counted and grouped into three clusters (first: impact, ventilation rate, and methane; second: variable, ammonia concentration, management, livestock; and third: artificial neural network, and prediction) have been highlighted by VOSviewer analysis; indeed, these groups of terms are all in accordance with the aims of this review. The most cited clusters (those with an occurrence value equal to 7) were variable, management, and artificial neural network.

### 3.2. ANN Applications to Livestock Production: Gases and Measurement Systems (RQ2)

The screening of the selected studies has highlighted how most of the applications of ANN have been focused on livestock sustainability by analysing the gases produced in a specific context. Most of the research studies included in this review predicted emissions (72%) and concentrations (27%), while one publication considered nitrogen excretion, expressed as kg/day [60]. In detail, NH_3_ has been investigated in 44% of the studies focused on predicting emissions [63,65,66,67,72,74] and concentrations [70,71,77]. Similarly, CH_4_ was considered in 22% of the papers, and the predictions were focused on emissions [62,75,76] and concentrations [69]. Minor gases analysed were CO_2_ and N_2_O. Specifically, Sun et al. [64] assessed CO_2_ emissions, Besteiro et al. [73] forecast CO_2_ concentrations, while Kolasa-Więcek [61] predicted N_2_O emissions. In two publications [60,68], emissions from manure were considered, although the gases involved were omitted.

Table 3 shows an in-depth analysis of the methods and devices involved for gas concentration measurement and emission estimation. Only two publications [69,74] applied ANN models to evaluate the sensitiveness of gas-monitoring devices and the effect of climate change on gases and cattle health, while most of the papers focused on methods, as shown in Table 2. Overall, regarding estimation approaches, mass balance was the prominent method used to assess emissions, accounting for 61% of the articles reviewed [62,63,66,68,70,71,72,73,74,75,76], while 11% used the CO_2_ equivalent method [61,67]. In detail, mass balance is based on a physics principle, where all input mass (i.e., feed intake, environmental and metabolic gases, bedding materials, manure storage, and treatments) and output mass (i.e., ventilation, manure management and removal, animal respiration, environment absorption) are computed to determine the final mass [78], whereas the CO_2_ equivalent method is a specific mass balance where gas mass is multiplicated to its global warming potential [79].

Most of the data recorded in the publications screened were measured by the researchers (i.e., 61%), while a significant part of the authors (i.e., 39%) applied data recorded in national/private databases or in previous studies, as shown Table 3. In all of the articles where datasets have been measured by the authors, the devices used in the trials were reported. The most frequently used device for gas concentration measurements was the Innova analyser, reported in 22% of the publications included in the review. In detail, Innova devices are based on the physical principle of photoacoustic spectroscopy, where absorbed lights are converted into sound waves. For climatic parameters, HOBO devices were the most commonly used data loggers. Concerning data gathering, 44% of publications declared measurement frequencies between 70 s and 24 h, while 39% of articles reported device calibrations, but only 33% of papers described measurement details (i.e., heights, sampling locations). Most of the publications have not specified any sampling frequency (67%). The main height interval is between 0.5 and 6 m, whereas the main locations are above the floor (i.e., no sensors in the pit).

### 3.3. Livestock Species Involved, Housing System, and Building Characteristics (RQ3)

Livestock farming is a multi-approach activity with different species, managements, and needs involved. Table 4 shows an analysis of the information on the animals (i.e., species, breed, and number of livestock), livestock building (i.e., housing system, and ventilation system), and barn management (i.e., manure parameters, manure management, and feeding) provided in the research articles analysed.

Cattle and swine are the most investigated livestock species in the reviewed literature, accounting for 33% of the selected papers. Two publications [61,68] considered a broader range (cattle, horses, poultry, sheep, swine, goats, donkey, mules, camels, and rabbits) of livestock species, one article reported trials involving poultry [64], while in three other papers [66,69,74], the considered livestock has not been specified. Among the studies investigated, only 33% of the publications have indicated the type of breed (i.e., Holstein-Friesian, Holstein crossbreed, Norwegian and Swedish Red, Yorkshire, and Large White x Landrace). In detail, three authors reported on the Holstein-Friesian breed [62,66,67], the study of Chen et al. [60] considered four breeds (i.e., Holstein-Friesian, Holstein crossbreed, Norwegian, and Swedish Red), while other authors [64,75] declared one swine breed (i.e., Large White x Landrace and Yorkshire), respectively.

**Figure 4:**
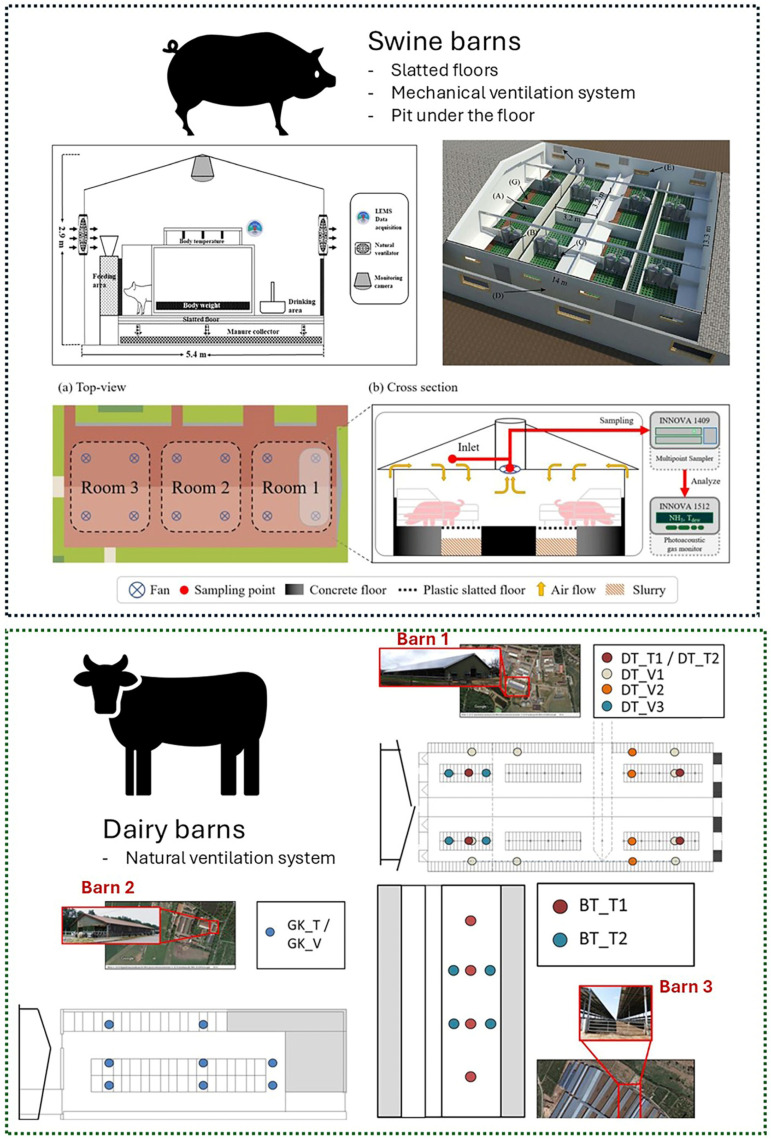
Layout of the livestock barns analysed in the reviewed papers.

Overall, only 61% of papers reported the number of animals involved in the trials performed. In detail, Lovanh et al. [63] conducted experimentations in a farrowing farm with 2000 swine, while Sun et al. [64] investigated ANN application in fattening facilities with a total of 960 animals. In Hempel et al. [66], the authors carried out tests involving 606 dairy cows, while Genedy et al. [72] assessed emissions from manure storage in a barn with 2680 cows. The numbers of animals varied, ranging from 18 to 2000 for swine and from 202 to 2680 for dairy cows. In Peng et al. [77], the authors applied an ANN model with data collected from 220 swine, while Shadpour et al. [62] investigated ANN application in a dairy barn with 202 cows. In Basak et al. [75], the authors carried out trails in a piglet farm with 18 animals. Most of the authors specified the housing type, although only a few research studies reported the barn layout for swine and dairy (Figure 4).

In detail, Hempel et al. [67] performed their experiment in a loose barn, characterised by lying cubicles with chopped straw and chalk as bedding materials and concrete walking. Other authors [60,72] carried out the investigations in a free-stall breeding environment characterised by cubicles with an adjacent exercise yard. In one study [62], the authors have not shared any information regarding cattle management. Overall, all swine-related buildings have been declared by researchers who also provide information on the housing system. In detail, in some studies [63,71], the experimental barn was a farrowing building characterised by indoor housing and a plastic slatted floor. Other studies [68,77] were carried out in fattening houses equipped with forced-ventilation devices and deep-pit manure storage. Another housing system analysed in some studies [64,70] was piglet houses, characterised by forced ventilation and a plastic slatted floor. In the screened papers, two ventilation systems were reported (i.e., naturally and forced ventilated). In detail, the prevalent ventilation system was forced [64,68,70,71,77], while natural ventilation was encountered in a minor investigation [66,67,72]. The remaining articles have not provided any information on the ventilation system involved in the investigation.

With regard to animal management, Table 4 showed that only the 22% of reviewed studies have provided information on feed composition. as well as manure parameters and manure storage have been reported by few studies.

### 3.4. Prevalent and Minor ANN Structures (RQ4)

**Figure 5:**
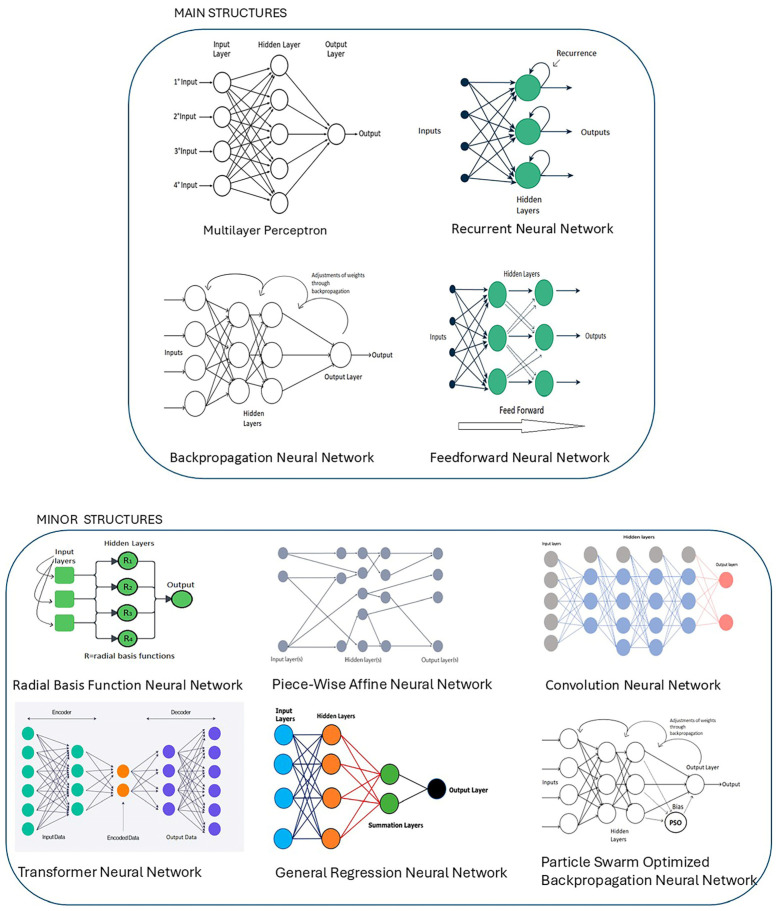
Structures of the artificial neural networks applied in the reviewed papers.

A study of the literature has revealed ten distinct ANN structures applied in the reviewed articles, summarised in Figure 5. These structures include Multi-Layer Perceptron (MLP), Feedforward Neural Network (FNN), Recurrent Neural Network (RNN), Convolutional Neural Network (CNN), Backpropagation Neural Network (BPNN), Particle Swarm-Optimised Backpropagation Neural Network (PSO-BPNN), General Regression Neural Network (GRNN), Radial basis neural network (RBF), Transformer Neural Network (TNN), and Novel Piecewise Affine (PWA).

MLP comprises interconnected nodes with at least three layers, forming a nonlinear mapping between input and output array numbers called vectors. Nodes are linked by weights, and their output signals, influenced by the sum of inputs and a nonlinear activation function, contribute to the model’s complexity [80]. FNN is structured with layers of neurons, each layer having connections (weights) to neurons in the preceding layer. Optimising, learning, and training an FNN involves identifying an effective network structure (function) and determining the weights. This process includes specifying the suitable neurons (activation functions) and determining their number, arrangement, and optimising the weight vector of the FNN [81]. RNN is a neural network model adapted to compute sequential data. Furthermore, this network has memory and can maintain information from previous steps of the training. RNN are more complex than MLP models and are deemed to be suitable for computer vision and image recognition tasks [82]. CNN models have convolutional operations features which merge two or more sets of data, especially grid-like, such as images. This structure is considered to be one of the most suitable to work on computer vision and face recognition [83]. BPNN models interact with datasets by applying a deepest-descent algorithm technique. This approach distributes each interaction in each variable, increasing the efficiency and the performance [84]. PSO-BPNN is a hybrid model that integrates BPNN with the PSO algorithm. As such, it computes weights as particle in a swarm, updating their position based on fitness [85]. GRNN is a memory-based model that does not require any training phase to solve computational tasks. This structure can filter outliers and wrong observations [86]. The RBF models have high convergence and learning speed and are suitable for recognition, approximation, and evaluation tasks [87]. TNN models have been proposed by Google^®^ and are based on attention mechanisms. This approach relies on related dependencies without regarding the distance between inputs and outputs [88]. The PWA model applies several combinations of affine (linear) functions to different regions of the dataset, each with linear transformation functions. PWA is generally found to work better compared to other models because the number of neurons is limited to the dataset’s boundaries [89].

In the literature, ANN structures have been modelled by describing the neurons inside each hidden layer. Each sequence of numbers shows hidden layers and neurons inside, e.g., 3–9–2 (three hidden layers with three, nine, and two neurons each). Input and output layers generally depend on the number of predictors and are not indicated in the models’ structure [90,91]. Overall, MLP structures are the primary structures considered by most of the papers selected in this review, accounting for 39% of the whole. BPNN contributed to 28% of the models, while RNN and FNN models accounted for 22% and 11%, respectively. Other models, such as PWA, CNN, GRNN, RBF, and TNN have accounted a minor contribution, mainly due to their inherent limitations. PWA and RBF approaches frequently face computational and performance challenges when dealing with large-scale datasets [92,93], while GRNN lacks the mechanisms to capture long-term temporal dependencies [93]. CNNs are less suited to modelling complex and multivariate emission dynamic [94], while TNN models require large datasets and need more computational efforts to be trained [95].

### 3.5. Dataset Characteristics (RQ5)

Dataset pre-processing is a common methodology widely applied in scientific papers. Most of the analysed authors (67%) considered pre-processing approaches such as min–max normalisation [60,69,74,76,77], Principal Component Analysis (PCA) [65,68], z-score [70], L2 normalisation [62], sliding windows [71], k-predictors selected by Statistica^®^ software [63], and Bayesian information criterion [64]. However, other authors have not given any information on whether they used a preprocessing method or not, and, if so, the type of preprocessing method used [61,66,67,72,75,76].

Data collected from devices and sensors generally require mathematical methods to be cleaned and useful for research applications. Normalisation is one of the most used approaches to achieve data standardisation, but the studies analysed did not apply a unique approach. In Basak et al. [70], the authors applied the z-score data normalisation technique to all numerical columns used in the model. In Genedy et al. [72], the researchers normalised the dependent variable (i.e., NH_3_ emissions) by using a natural logarithm, whereas sine and cosine functions were utilised for the independent variables (i.e., time, temperature, wind speed, and wind direction). In Hempel et al. [67], the authors normalised the input vector to obtain a mean equal to 0 and the standard deviation equal to 1. In fact, ANN structures tested on normalised input data have obtained lower errors and accuracy loss [96]. Generally, ANN algorithms perform well with nonlinear and complex datasets without any prior assumption, even if datasets are imprecise and noisy [73]. On the other hand, the authors found how outlier treatments can influence the prediction [72]. In fact, in their study, checking and removing outliers produced results for the emission estimation by using linear regression and short measurement periods. Nevertheless, most of the studies analysed did not add specific information on outlier treatments.

**Figure 6:**
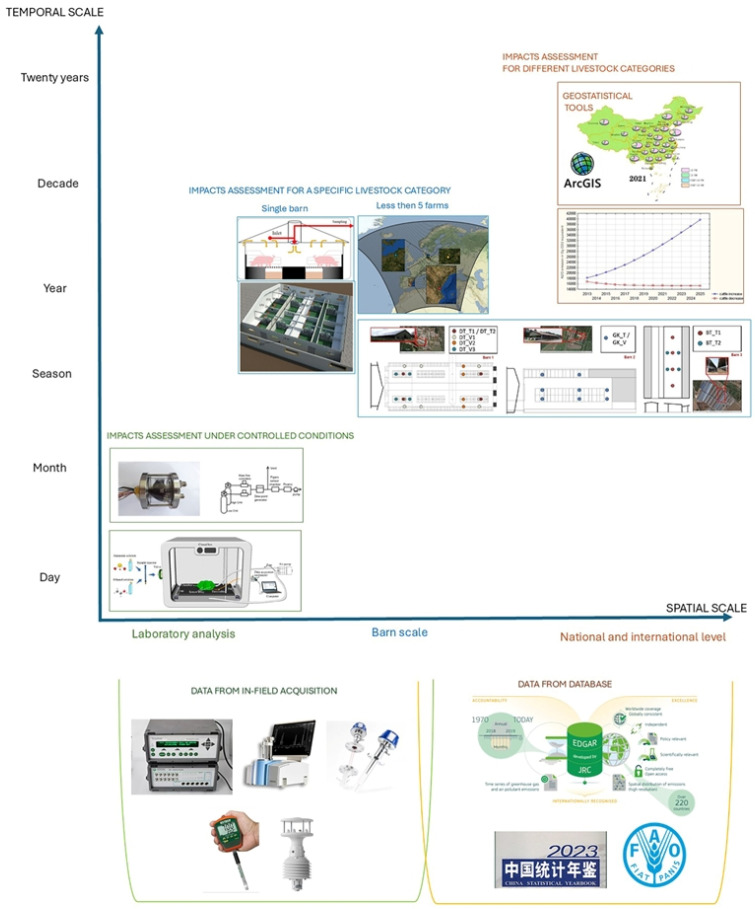
Overview of the temporal and spatial scale of papers analysed related to data acquisition.

**Figure 7:**
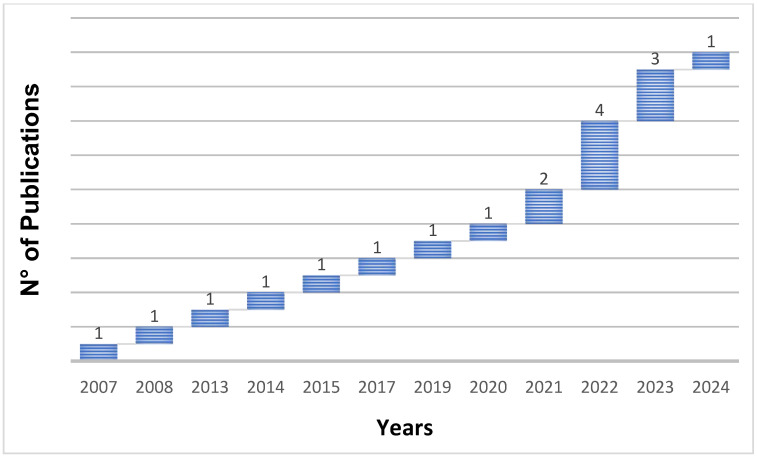
Overview of the temporal evolution of the publications screened.

The length of data acquisition has been found to be very different in the articles selected in this study. Several experiments were conducted with different temporal intervals (Figure 6). In detail, 44% of the papers analysed data for 1 year or less. In another 44% of the articles, data was collected in over 1 year of measurements, up to 26 years [60]. Yet 1 of the 18 articles did not provide the period of the investigation [69]. Therefore, the research studies analysed can be assessed by considering the multi-temporal, multi-scale, and location data in the acquisition, as reported in Figure 5, which show that, after increasing the scale, monitoring accuracy decreases. The temporal evolution of the articles highlights a constant presence of the ANN models applied in livestock sector investigations. However, a peak in publications was observed during the early–mid years of the current decade, showing an increasing interest in the topic (Figure 7).

Another crucial aspect of the input dataset regards the different variables included. To facilitate the classification of variables related to the aims of the studies, input variables have been reported in Table 2. Based on the input variables, some authors have directly measured the outputs they wanted to predict while others have indirectly estimated the outputs. Regarding direct evaluation, in detail, in this publication, the authors assessed the direct emissions of N_2_O from agricultural soils in Poland by considering the following input variables: pastures, meadows, arable land, crops, and livestock population [61]. Other articles [64,73] used environmental parameters (i.e., temperature, pressure, and water vapour) and CO_2_ as input variables to forecast CO_2_ concentrations. Similarly, in a recent publication, the authors predicted NH_3_ concentrations and emissions by using the following input variables: NH_3_ concentrations, ventilation rate, temperature, and relative humidity [71,77]. The input variables used by Stamenkovic et al. [75] to forecast CH_4_ emissions expressed as kg per capita were livestock populations, waste deposit, gross domestic production, and primary CH_4_ production.

Indirect output estimations have been applied by several authors. In detail, several publications have selected input variables related to livestock management [60,61,70,76] (i.e., animal growth cycle, rearing cycle, commercial scale husbandry coefficient, mass, age, feed intake, number of animals, milk yield, dietary forage proportion, nitrogen dietary intake, diet metabolizable energy content, age at calving, fat yield, and protein yield) and excreta [66,68] (i.e., manure storage level, excreta rate, manure moisture, pH, and temperature) and examined them in relation to environmental parameters (i.e., time of day, season, ventilation rate, weather conditions, temperature, and wind speed and direction) to estimate gaseous emissions [72]. In Lim et al. [65], the authors took into consideration the following variables: temperature (i.e., measured at the floor surface, at 0.5 m, and at 1.5 m above the waste lagoon), pH, moisture, pressure, wind speed, and relative humidity. In Hempel et al. [67], the authors considered soil types, weather conditions, manure characteristics, agronomic factors, and measuring techniques as input variables to estimate NH_3_ emissions. In other studies [64,73], the authors estimated emissions as CO_2_ equivalent for livestock units based on the input environmental variables (i.e., temperature, relative humidity, sea level pressure, radiation, zonal and meridional wind, wind speed and direction) and hourly emission derived from ventilation rate, time, temperature, wind speed, and direction.

### 3.6. Evaluation Metrics Applied (RQ6)

A set of 13 evaluation methods has been identified in the research articles (Table 5). The authors of the selected studies have often utilised more than one quality metric, among the following: MAE, RMSE, Accuracy, R^2^, Total absolute error (TAE), MSE, Index of Agreement (IA), Mean Absolute Relative Error (MARE), Residual Prediction Deviation (RPD), CCC, Pearson Correlation Coefficient (PCC), Standard Deviation (SD), Standard Error Prediction (SEP), observations standard deviation ratio (RSR), and Average Absolute Percent Relative Error (AAPRE).

RMSE emerged as the prominent evaluation metric, accounting for 67% of the examined studies and showing its main role in ANN applied in livestock production in all the studies screened. R^2^, accounting for 55% of the publications, have been used in model quality evaluation tests. MAE has been considered in 39% of the analysed studies, being among the three most used evaluation metrics, while MSE accounted for 11% of the papers [65,67]. The remaining validation methods to evaluate models were TAE by [72], SEP, SD, RSR and APPRE by [69], IA by [75], CCC by [60], PCC [76], MARE [64], and RPD by [76].

Based on the reviewed papers, 67% of the researchers have evaluated their models by implementing two or more quality metrics. The most used combination of evaluation criteria, compared to other combinations, was RMSE and R^2^ (39%), showing the authors’ preference in evaluating the quality of the models tested.

### 3.7. ANN Training Algorithms Applied (RQ7)

Training algorithms (TAs) are computational procedures that control and assess the size of the weights according to the data. Weights are mathematical parameters that show the strength or importance of the connections between neurons. Each time an input variable is computed by neurons and is multiplied by the corresponding weight [97]. The aim of TA application is to teach the ANN model how to make predictions or decisions based on input data [98]. A relevant feature to considered in TA application is convergence that represents the value of training loss during computational work of ANN models. When convergence speed decreases, the training process is stable, and the ANN can make accurate predictions [99]. In the literature, the number of times samples from the dataset are computed by the training algorithm (epochs) has been established as hundreds or thousands and deemed sufficient to reduce the training error [100].

Five different training algorithms have been applied in the research studies analysed: backpropagation (BP), Levenberg–Marquardt (LM), Bayesian regulation (BR), Broyden–Fletcher–Goldfarb–Shanno (BFGS), and scaled conjugate gradient (SCG) algorithms. BP is a descent gradient computational algorithm able to minimise error during the process of training. Such minimisation is obtained by adjusting the weights of the ANN through error computation made in previous iterations [101,102]. The LM method represents a regularisation technique coupled with the Newton method for solving nonlinear equations. The algorithm updates the model parameters iteratively to minimise the loss, incorporating both the gradient information and the curvature of the objective function [103]. SCG method is an iterative algorithm primarily utilised for numerically solving systems of linear equations with positive-definite matrices. It is particularly useful for large sparse systems encountered in scenarios such as partial differential equations and optimisation problems [104]. BR training algorithm offers increased robustness compared to standard back-propagation nets, e.g., MLP and FNN, potentially eliminating the need for extensive cross-validation. When utilising BR, ANNs transform nonlinear regression into a statistically well-posed problem, resembling ridge regression and making the validation process unnecessary, which contrasts with the scaling in traditional regression methods such as back propagation [105]. BFGS is an iterative method that minimise, or maximise, functions. Starting from an initial point, it estimates the inverse Hessian matrix, which is the identifying matrix, and, at each computation step, the algorithm calculates the gradient of the function [106]. Based on the literature analysis, BP algorithms are the most used to assess ANN models in livestock productions (i.e., 55% of the studies analysed as shown in Table 2). BGFS algorithm showed a minor contribution accounting for 17% of the screened articles as well as LM, BP, and SCG have been applied in 11% of the articles selected. However, some articles have not specified any of the training algorithms considered [71,72,73].

### 3.8. Comparisons Among Methodologies Based on Statistical, ML, and ANN Models (RQ8)

In the 18 articles selected, the authors applied various methodologies, evaluating different ANN models [61,62,63,64,68,69,71,72,74], statistical and ANN models [66,68,75,76], statistical, ML, and ANN models [60,66,67,70], and the performance of ML and ANN models [77]. The comparison between different ANN models is the most prominent methodology applied by the researchers, accounting for 50% of the reviewed publications. In detail, Kolasa-Więcek [61] has applied several hundreds of MLP models. The best performance (R^2^ = 0.99 and 0.98) has been obtained by two MLP models with three hidden layers with 16–5–1 neurons and a MLP 9–4–1, respectively. In the study of Martinez et al. [62], the authors modelled several ANN structures to assess CH_4_ concentrations; however, only MLP 5–14–19–1 was fully reported with a performance of RMSE = 0.2. Four different MLP structures (i.e., 5–13–1, 5–6–1, 5–7–1, 5–15–1) have been modelled in Lovanh et al. [63]. The best model was 5–7–1 MLP that have performed MAE = 0.01, MSE = 0.01, and SD = 0.03. In Sun et al. [73], the authors carried out trials based on RBF model application, but no structure information was specified, except for the reported R^2^, ranging from 0.99 to 0.81. MLP models tested by Küçüktopcu and Cemek [69] to estimate CO_2_ emissions consisted in 1 hidden layer with 8 to 15 neurons, obtaining R^2^ values ranging from 0.994 to 0.998, RMSE from 1.041 to 1.982, SEP from 1.712 to 3.259, RSR from 0.050 to 0.095, and AAPRE from 4.173 to 10.862 in the various tests carried out where authors changed and tuned training algorithms. In Park et al. [71], the authors assessed MLP (undefined structure), RNN (3 hidden layer with 64 neurons), CNN (1D), and TNN (structure undefined) models. Performance, expressed as MAE, was as follows: MLP ranged from 2.15 to 2.24, RNN ranged from 1.78 to 1.95, CNN ranged from 1.87 to 2.02, and TNN ranged from 1.73 to 1.90. In Genedy et al. [72], the researchers evaluated RNN models to estimate NH_3_ emissions but there were no definitions of applied structures. According to the authors’ statements, MAE and RMSE ranged from 0.97 to 2.19 and from 1.64 to 3.34, respectively. In Besteiro et al. [64], the authors applied FNN models to predict CO_2_ concentrations, where the number of layers (3) have been reported, but no information on the number of neurons have been declared. The models’ performance was RMSE = 26.33, MARE = 1.26%, r = 0.99, and IA = 0.99. In Shi et al. [74], researchers have assessed BPNN, PSO-BPNN and RNN models to estimate NH_3_ emissions. Based on the outcomes of the tests performed, BPNN obtained better performance (R^2^ = 0.98, MAE = 9.6, and RMSE = 14.25) compared to RNN (R^2^ = 0.94, MAE = 15.2, and RMSE = 24.5) and PSO-BPNN (R^2^ = 0.98, MAE = 8.42, and RMSE = 14.56).

A minor contribution (26%) has been made by researchers to the comparison between statistical and ANN methodologies. In detail, Lim et al. [65] have applied PWA model (10–26–2) and MLR approach. ANN outperformed MLR, showing better value of R^2^ (0.99 vs. 0.66). Recently, He et al. [68] carried out tests by using BPNN and statistical (ARIMA) models to estimate manure emissions. However, no information regarding hidden layers and neurons has been given by the authors. BPNN scored a range of RMSE values from 0.93 to 0.98, while ARIMA scored a range of RMSE values from 6.89 to 8.35. In Basak et al. [70], the authors applied three statistical methodologies (i.e., MLR, RR, and PR), ML (RF), and FNN to estimate CH_4_ emissions. The results obtained showed that the models had similar performance (MLR: R^2^ = 0.90, RMSE = 0.01; PR: R^2^ = 0.91, RMSE = 0.01; RR: R^2^ = 0.92, RMSE = 0.01; RF: R^2^ = 0.97, RMSE = 0.01; BPNN: R^2^ = 0.90, RMSE = 0.01). In Stamenkovi et al. [75], the authors assessed statistical (MLR) and ANN models (i.e., BPNN and GRNN structures, yet undefined) to estimate CH_4_ emissions. The GNRR model showed similar results (IA = 0.97, MAE = 3.6, RMSE = 7.0, PCC = 0.94) compared to BPNN (IA = 1.00, MAE = 3.4, RMSE = 5.0, PCC = 0.97) while MLR’s performance were slightly worse (IA = 0.83, MAE = 11.3, RMSE = 14, PCC = 0.75). In Shadpour et al. [76], the authors evaluated MLP and statistical (PLS and ANOVA) models to estimate CH_4_ emissions. Better predictive results have been obtained by MLP models (ranging from 0.93 to 0.97 for RMSE, 1.06 to 1.10 for RPD, 0.320 to 0.360 for PCC) compared to ML structures (PCC = 0.255, RMSE = 90.45, RPD = 1.21).

Other authors who have applied various kinds of methodologies (statistical, ML, and ANN), accounting for 16% of the screened articles. In Hempel et al. [66], the authors used MLP models with one to three hidden layers, ML models (RF and SVM), and a statistical model (LR). MLP performance have been reported (with R^2^ ranging from 0.56 to 0.85), while ML and statistical performance were undefined. Similarly, in Hempel et al. [67], several MLP structures (from 1 to 10 hidden layers and 4, 8, 16, and 32 neurons), ML models (XGBoost, SVM), and a statistical model (LR) were evaluated in 27 scenarios to estimate NH_3_ emissions; each scenario was associated with diverse socioeconomic, technological, and political developments, resulting in varying gas concentrations. The best performance was obtained by the 7th scenario (MAE = 0.480, RMSE = 0.418, R^2^ = 0.088) while the 13th scenario scored the best TAE (0.015). In Chen et al. [60], the authors evaluated different statistical (MLR), ML (i.e., RF, and SVM), and ANN approaches to evaluate nitrogen excretion. The outcomes were similar among the methodologies proposed (MLR: RMSE = 44.7, CCC = 0.60; RF: RMSE = 46.8, CCC = 0.58; SVM: RMSE = 44.9, CCC = 45.3; FNN: RMSE = 34.7, CCC = 0.70). Only one publication underwent a comparison between ML and ANN models [77]. In this paper, the authors applied ML (i.e., SVM, and XGBoost) and undefined ANN (i.e., BPNN, and RNN) structures. Overall, the models’ performances were similar (RNN: R^2^ = 0.92; BPNN: R^2^ = 0.80; SVM: R^2^ = 0.89; XGBoost: R^2^ = 0.92; PSO-RNN: R^2^ = 0.96).

## 4. Discussion Overview

### 4.1. The Role of ANNs in Concentration and Emission Estimation

This study reviewed 17 years of publications of ANN applications and approaches, addressing gaseous emissions in livestock production. In the last four years, the number of publications coupling gaseous emissions from livestock housing and ANN has increased, contributing to identification of new tools for researchers in the field of precision livestock farming. The distribution of studies across 27 countries demonstrates a widespread global interest, and recognition of the importance of research on livestock emissions. This reflects a collective international effort to address environmental challenges associated with livestock production. Most of the research on livestock emissions has been conducted in Europe, indicating a strong focus and investment [107] in environmental and agricultural research within the continent. In fact, many European countries are developing research to refine the estimation of emission factors from livestock farming [51,108], and, in the meantime, improving protocols and regulations aimed at monitoring, assessing, and reducing gas concentrations and related emissions [109,110]. In addition, cattle farming is the most prominent in Europe due to related economic, social, and agricultural impacts [111], and this importance is confirmed by the publications reviewed (7 out of 18 papers’ case studies located in Europe). Asia, being the second-largest contributor, highlights the region’s growing involvement and concern in addressing livestock emissions, potentially driven by rapid industrial and agricultural development.

The examination of ANN architectures revealed an interesting variety of applications. The ANN models enable real-time prediction, rapid responses, waste reduction, automation, enhancing efficiency, problem detection, and management of health conditions in livestock related to animals and farmers. These tools could have several fields of applications in the PLF, and, in detail, the main aspects of the PLF reported in different studies [112,113] encompassed health, welfare, and production, as well as animal behaviour, environmental barn conditions, and their effects on the three pillars of environmental, economic, and social sustainability. The PLF could benefit from ANN models’ applications, not substituting humans in decision-making, but supporting the process of decision-making for both farmers, policymakers, and stakeholders. For instance, in Stamenkovic et al. [75] were applied ANN model to forecast CH_4_ emissions at national level, opening the opportunity to apply this modelling technique to emission inventories with the consequent implementation of environmental management actions. The main advantage of the ANN models’ application would be the reduced number of input parameters compared with conventional emission inventory-based models. In predicting emissions and concentrations from livestock housing, most of the reviewed papers concentrated on NH_3_, followed by CH_4_, whereas minor interest was devoted to CO_2_ and N_2_O (derived from agricultural soils connected to livestock production). NH_3_ and CH_4_ emissions represent the main gases emitted from livestock sector. Indeed, manure management from livestock is responsible for about 42% of the total NH_3_ emissions in Europe, whereas CH_4_ from manure management and enteric fermentation contributed 53.4% of emissions from livestock to the total CH_4_ emissions in Europe [114]. In this context, NH_3_ emissions are the most investigated due to the availability of both high-cost, mainly utilised by scientific research as reference, and low-cost technology implemented and assessed to provide farmers with an economical solution for monitoring purposes [115,116,117]. CH_4_ emissions are also investigated, but to a lower extent, since nowadays there is a lack of low-cost sensors for measuring CH_4_ concentrations. In fact, current technologies use sensors for CH_4_, but their measurement range is not compatible with concentrations recorded in the barns and, consequently, data are not always complete. In the literature, Martinez et al. [62] have analysed low-cost sensors for CH_4_ measurements combined with ML, but the tests have only been carried out in laboratory conditions. Therefore, considering the applications of low-cost sensors and small dataset, the combination of ANN and specific data acquired by using different devices could improve knowledge on emission factors and concentrations characterising each livestock farm.

### 4.2. ANN Characteristics and Datasets Applied

**Figure 8:**
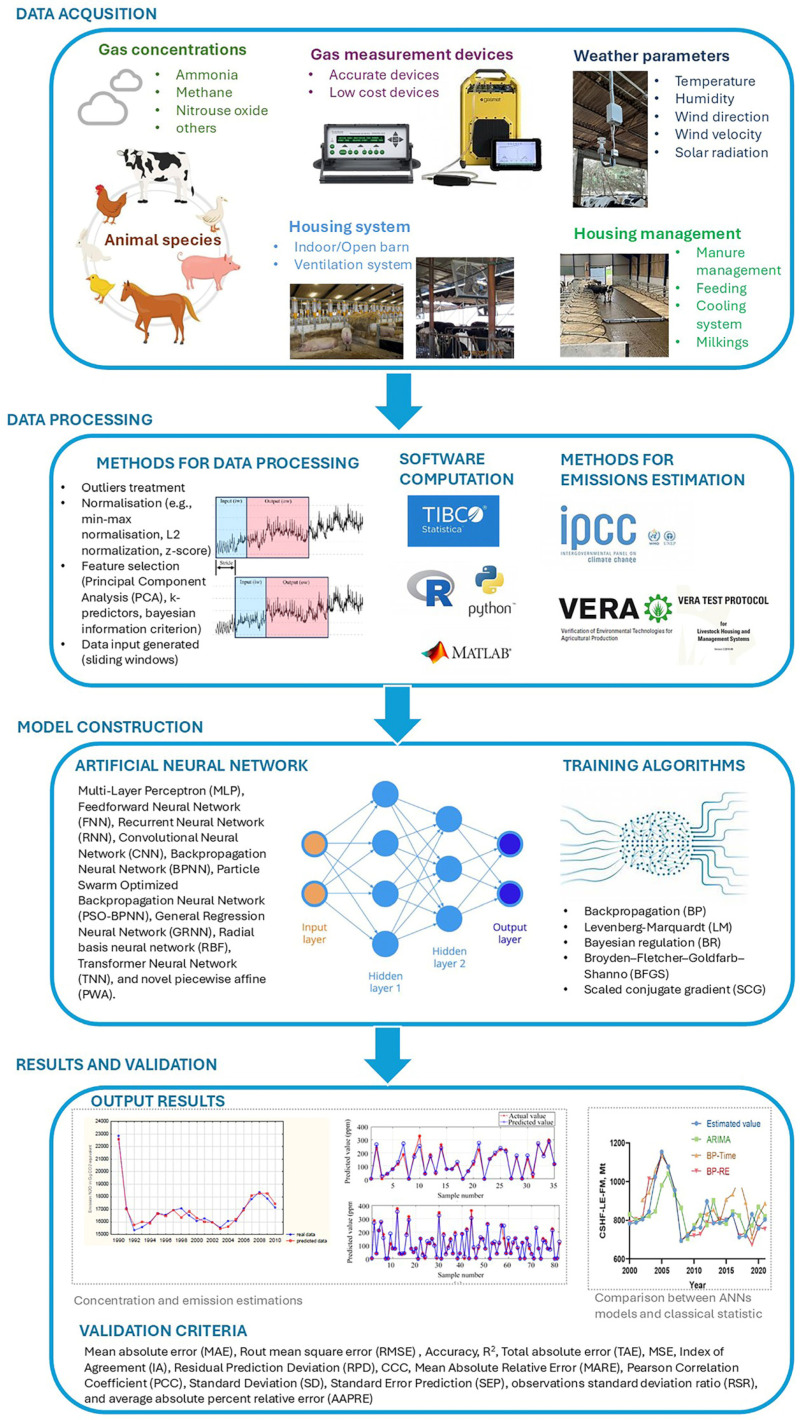
Flowchart for analysing concentrations and emissions from livestock farms.

In the context of livestock farming, the application of ANNs have allowed for many challenges for researchers. Figure 8 provides an overview of ANN application from data collection to data estimation, with a focus on data processing and modelling approaches.

Although ANNs have demonstrated significant potential in improving prediction accuracy, currently there are several critical issues to be addressed. The availability and quality of dataset are often affected by the use of heterogenous protocols, which reduces comparability across studies. Measurement methodologies add uncertainty, since different sensor technologies and approach introduce systematic bias [15]. In particular, data measurements and availability are of the utmost importance for improving model accuracy. Increasing the size and the availability of training data [118] and removing outliers and bias are the first steps to improve the performance of ANNs [119]. Among the articles analysed, several of them (9 out of 18) had a limited experimental period or highlighted a limited set of data for emission estimation. For instance, emission factors, based on annual data (VERA protocol) and concentrations, are influenced by seasonal conditions. Most of the papers selected directly measured data, while a minor number of articles utilised data collected from existing databases. Publications based on this kind of database have thus not specified any devices involved in the case studies, while authors who collected data declared the instruments used in the trials. As a consequence, data quality, depending on instrument accuracy, cannot be considered in these studies. Other important aspects (i.e., frequency of measurements, calibration of the instruments, and measurement deployments) of data collection are absent in most of the articles selected, making difficult to reproduce these approaches in further studies. In detail, sensors placement is crucial to understand the complex dynamics of the livestock gases due to the relevant differences among them. The distance from the direct source reduces both accuracy and reliability of the measurements [120,121]. Emissions are subject to significant variability, influenced by multiple factors. Therefore, sampling frequencies play a key role to capture and understand short-, medium-, and long-term dynamics [122]. To ensure robust experimentation, researchers should establish a clear protocol that specifies the sensor selected for the selected task, sampling conditions, and distance from the source, which are a prerequisite for generating valid data [121].

ANN models showed an improvement with larger datasets, indicating their potential scalability and robustness when managing more extensive data. In detail, ANN models significantly outperformed ARIMA models, demonstrating a high coefficient of determination and lower prediction error [73]. ANN models performed slightly better than PLS models, but the difference in RMSE was not substantial [76]. RF (ML model) outperformed statistical and ANN models overall. However, ANN models performed better with larger datasets [70]. ANN models significantly outperformed MLR models, showing higher indices of agreement and R^2^ values [75]. ANN models outperformed MLR models in predicting nitrogen emissions, with lower RMSE and higher concordance correlation coefficients [60]. ANN models outperformed MLR models, achieving much higher R^2^ and significantly lower MSE [65]. The need of having a large dataset has been specifically investigated in several studies analysed. For instance, Park et al. [71] identified good performance with RNN, CNN, TNN, and indicated the minimum size of the dataset. These models allowed for accurate predictions by using a minimal number of measurements, independent of the size of facilities and environmental conditions. In detail, the data input of two days is sufficient for medium-to-long-term prediction over several weeks. In the study of Hempel et al. [66], increasing the measurement time from one week to two weeks provided only a slight improvement in the prediction performance. In general, it has been found that almost all best-performing scenarios had six measurement periods of at least one day, in line with the VERA protocol [109].

In addition to the quantity of data, it is relevant to consider the number of variables that affects the efficiency of the ANN applied. In fact, the selection of an appropriate set of input variables contributes significantly to the model’s development [64]. Based on a sensitivity analysis, some studies identified the most significant variables that affect the emissions (i.e., dependent variables). For instance, Kolasa-Wiecek [61] identified that the amount of nitrogen fertiliser was the variable that influenced the direct emissions of N_2_O from soils (i.e., output variable), followed by livestock housing (i.e., cattle, horses, poultry, sheep, swine, goats). The study by Shi et al. [74] highlighted that there are several factors affecting concentrations and emissions (i.e., physical characteristics of the site, indoor environment, climatic conditions, and barn management), yet it could be demonstrated that a reduced number of factors were able to describe the main variability. The use of the PCA combined with ANN could then be helpful in defining fewer uncorrelated variables to the ANN and reducing the number of variables. In this way, model complexity is reduced, as well as computational cost, overcoming problems related to overfitting. Moreover, the precise choice of the input parameters would determine an improvement of the model performance. Also, in Peng et al. [77], the authors concluded that adding environmental parameters increased the accuracy in predicting NH_3_ concentrations compared to models with only NH_3_ as input. However, the accuracy of the model decreased after adding too many environmental parameters (eight instead of four). In conclusion, most of the tests carried out in the publications selected, have applied several input variables between four and eight, while a minor contribution has been performed with more than eight input variables.

To enhance prediction accuracy, the models found in the literature considered multiple factors, including the type of building, ventilation system, weather parameters, diurnal and seasonal effects, animal-related factors (i.e., species and breed), and barn management, as well as manure characteristics [117,118,119]. Indeed, the dependence of gas levels on these kinds of factors has already been highlighted in several studies based on classical statistical assessments. However, statistical models proposed in this field [123,124] were not usually built to perform predictions, with the exception of the study by [19], where the outcomes suggest that ML models (ANN and RF) consistently outperform statistical models, across various studies, in predicting livestock emissions, showing higher R^2^ values and lower RMSE or MSE values.

While traditional statistical models like MLR, PR, and PLS provide quite reliable results, they generally lack in accuracy and reliability compared to the ML and ANN models. For instance, Chen et al. [60] reported that ML algorithms might be a better alternative to classic statistics to develop models for predictions from cattle farms because ML can explore datasets and understand correlations between inputs and outputs. However, this cannot be considered a fixed rule, since in some studies [70,125,126] it was found that ANN models did not significantly improve prediction accuracy compared to statistical models. In some cases, statistical models performed better, such as in Stamenkovic et al. [75], where regression-based models performed even better compared to the ANN models. Also, in Hempel et al. [66], a higher variability among different sampling strategies was found with an ordinary linear regression. The comparison among ML and classical statistics, as well as comparisons between the performance of different ML models, have been helpful to define appropriate parameters and algorithms specifically tailored to evaluate NH_3_ concentrations and emissions [60,77]. This has often been a difficult task for researchers, since gas concentrations in barns are mixed, complex, and usually have nonlinear dynamics depending on both environmental factors and barn management, and, moreover, these factors vary from region to region.

### 4.3. Future ANN Applications and Research Needs

Based on the literature, attempts have been made to provide knowledge on emissions from livestock farming by comparing different ML models analysed in this review. Despite the progress that has been made, challenges persist, including incomplete or inconsistent dataset information (i.e., pre-processing, timeframe, parameters), ANN structures, validation criteria, and training algorithms. This review highlights how there is no unique methodology to approach emissions predictions. In general, in the context of gaseous concentrations and emissions from livestock farming, it is possible to provide some hints for future ANN applications. In detail, MLP models have emerged as the prominent structures applied in livestock production to assess gas emissions and concentrations in most of the publications. This type of ANN has obtained the best performance compared to other models selected in this review (i.e., FNN, RNN, CNN, RBF, PWA, BPNN, PSO-BPNN, TNN and GNRR). It is worth remarking that, among time-series investigations, RNN emerged as the prominent in the screened literature, demonstrating their capability to address prediction challenges in the livestock sector. Furthermore, two prediction horizon approaches were applied: the one-step and the multi-step. The one-step approach was used to evaluate the capacity of the model to predict emissions values in a real-time acquisition [74,77], while multi-step approach focused on forecasting values over extended horizons based on the data collected [71,72].

However, in the literature, other ANNs models are applied, such as Hybrid ANN (HANN). Although HANN models reduce computational time and accuracy loss [127], their performance has not been investigated yet for concentrations and emissions prediction. Therefore, further investigation is needed to evaluate their potential for assessing livestock sustainability. In the screened papers, two studies [71,77] have applied a hybridisation of two ANN models, consisted in connecting two different ANN models to obtain better performances. In detail, in the study by [77], HANN obtained better performance compared to non-hybrid ANN, whereas in the study by [71], HANN assessed NH_3_ emissions, with slightly worse validation scores compared to non-hybrid ANN. However, these models have not been treated as different models in this review.

In most of the articles (11 out of 18), no details have been presented by the authors about the numbers of hidden layers and/or the neurons allocated to each layer. Such omissions reduce the contribution to the creation of ANN models capable to overcome livestock sustainability challenges because those models cannot be reproduced in further studies. Indeed, the input and output variables of the model determine the number of neurons in the input and output layers, as stated by [68]. It is, however, a consolidated practice that the number of neurons in the hidden layers is determined by trial and error, as there is not a specific procedure.

Training algorithms have played a key role in the process of creation of ANN models. BP emerged as the principal algorithm applied in the scientific articles analysed in this review, whereas the LM, BR, BFGS, and SCG algorithms gave a lower contribution in the literature screening. According to the publications selected, BP has shown reliable performance and adaptability in livestock production to assess predictions of gaseous emissions. However, no comparison between BP and other training algorithms have been conducted by the authors presented in this review. In addition, researchers have validated their models by using RMSE and R^2^, preferably implementing two or more validation criteria to make comparisons with other studies in the literature. New research studies could explore the performance of different training algorithms to evaluate the suitability of the model with the concentrations and emissions predictions, validating the results with two validations criteria.

The literature analysed did not include predictions based on data derived from recent technologies and simulation models (e.g., IoT, low-power networks, and computational fluid dynamics—CFD). Indeed, several studies have shown the potential synergies between technologies, such as remote sensing, cloud database, and farming automations [73,128,129], and potentialities of data acquired by utilising these new technologies. They have been recently introduced in livestock farming to support various activities, being suitable to enhance animal health [20,128], reduce environmental impact, and lower energy consumption [116,117,128,129,130]. However, these technologies need to be totally interconnected to cloud servers to work properly, yet these servers are often rented for a fee. When farmers could not afford such costs, this issue could lead to a constraint in the application of these technologies [129]. Simulation models such as CFD are valuable approaches to understand spatiotemporal dynamics; they have been applied in the livestock sector to assess the complex matrix of the environment and the distribution of gases [131,132,133]. On this basis, ANN predictions could take advantage of these new technological and modelling advances to assess modern data acquisition tools and take into consideration simulated dynamics within the livestock barns.

Identifying key variables is a pivotal point of future research on ANN models. Selection of appropriate variables is recommended as they can influence performance, applicability, and accuracy of the ANN models. These variables should include, for instance, animal physiological indicators, environmental factors, and management practices, which can impact models’ predictive performances. In the papers analysed, although most of the authors have declared the species involved in their trials, only a few papers have specified the breeds. Since each breed has different enteric fermentation, producing variable pollutant gases [134,135], neglecting that these data could negatively influence ANNs construction. Another relevant parameter is the number of animals involved in the studies selected. Most of authors have applied an average of 800 animals per test, while other publications have applied few animals (6) or a very high number of animals (2680). This parameter is quite relevant for outcome accuracy because the production of gases is influenced by the number of animals [136]. Other aspects that should be included when dealing with livestock emissions are livestock management, type of housing and ventilation system, feed intake and composition, and manure management. However, many of the research studies analysed have omitted these crucial details, reducing their contribution to livestock research due to the lack of information; without these details, an experiment is not representative of the complexity of the livestock environment [137,138]. Furthermore, in the context of livestock applications, ANNs should include the description of livestock categories (e.g., species, breed, age, and number of animals), environmental conditions (e.g., temperature, humidity, and ventilation), housing system and building typology (e.g., free-stall, tied housing, and open/closed barn), cooling system (e.g., presence of fans or sprinklers), and barn management (e.g., herd management, microclimate conditions, and manure management). For instance, the details of animal feeding should be considered, since enteric fermentation is correlated with pollutant gas production [139,140]. Similarly, manure management and composition have been mostly neglected in the papers screened, although their contribution to pollutant emissions is well known [138,139].

Very different emission factors have been computed for various livestock farming conditions. For instance, in cattle barns, NH_3_ was influenced by the housing system (i.e., tied stall, and loose housing system with cubicles and solid floor or perforated floor), with tied housing having a lower NH_3_ emission factor (6.9 g LU^−1^ d^−1^) compared to loose housing (29.3 g LU^−1^ d^−1^) due to minor soiled area covered by faeces [118]. With regard to barn functional areas, feeding alley have shown the major contribution of NH_3_ emissions (ranging from 12.55 to 62.15 mg m^−2^ h^−1^), while the resting area have contributed with a minor impact (from 2.86 to 4.61 mg m^−2^ h^−1^) [140,141]. In detail, specific housing systems with different floor types and manure-handling systems (i.e., perforated floor, scraper on concrete floor, flushing system, scraper on rubber mat), as well as bedding cubicles (i.e., rubber mat cubicles, straw cubicles, rubber mat cubicles and straw), influence the emission release in the barn.

Also, ANNs have proven to be a useful tool to predict emissions related to barn mitigation strategies [142,143,144,145,146]. These mitigation strategies have been investigated to reduce the impacts of pollutant gases and the best combination of approaches to mitigate NH_3_ emissions in dairy farms [118] were the following: scraping combined with manure acidification (up to 44–49% reduction in emissions), solid flooring combined with scraping and washing (between 21 and 27% reduction) and floor scraping combined with washing and floor scraping only (17–22% reduction). Environmental impact evaluation of livestock emissions, based on life cycle assessment (LCA), from the acquisition of materials to production, highlighted that enteric fermentations have obtained the highest contribution, accounting for 38.37% of the total livestock emission [79].

In the literature, protocols and research projects (e.g., VERA, and DATAMAN) have proposed a classification of relevant parameters that could be acquired during a measurement campaign to characterise emission factors computed for a specific animal category. These data should be systematically recorded and integrated into the model to enhance its accuracy and practical usability. Only a few studies analysed in this review have used protocols for data acquisition or can be considered sufficient for livestock characterisation. Overall, some studies lack on information about ANN applications, and some others lack information on livestock farm features. It is crucial to highlight the importance of database accessibility for all stakeholders. The research community is increasingly acknowledging this need through the inclusion of data availability statements in scientific publications, encouraging researchers to share data in order to have higher transparency as well as create opportunity for further advancement in the field.

The development and application of ANN models in livestock farming requires a multidisciplinary approach, combining computational methods with animal science expertise. In this context, Agricultural Engineering plays a central role, bridging the gap between data-driven techniques and domain-specific knowledge. Agricultural engineers are essential to designing, training, and validating ANN models that accurately reflect the complexity of livestock systems. This interdisciplinary synergy is crucial for developing reliable decision-support tools that enhance productivity, sustainability, and animal welfare. Future research should focus on refining ANN models through closer collaboration between disciplines and by adopting validation protocols that reflect real-world farming conditions.

## 5. Conclusions

This study highlights the research advances in the prediction of concentrations and emission estimation to improve livestock sustainability, providing a detailed framework of ANN livestock applications. This review analyses the role of ANNs, their main features, and the datasets applied to achieve substantial improvements in prediction accuracy. The results show that ANN models can be applied to a wide range of scenarios, including concentrations and emissions from different livestock farms with specific animal species, housing systems, and animal and manure management. In addition, ANNs showed their potentiality in this field, where applications from a single-barn-scale to a specific territory (e.g., countries, and regions) have been explored. Currently, ANNs based on the data derived from in-field measurements and acquired by specific measurement devices have higher prediction accuracy than ANNs that applied global databases. In this latter case, studies that analyse the environmental impacts on a large scale generally predict emissions by using emission factors that are not accurate. Indeed, this emission factors do not consider the main features of livestock barns discussed in this review. Consequently, emission predictions, though generic, are applied to a specific country or region. Regarding this issue, the role of ANNs has high potential, as gas concentrations and emissions can be predicted with high accuracy, starting from specific in-field measurements. The ANN models could improve emission factors for specific livestock categories and barn management, increasing knowledge in this field. This knowledge could represent the starting point for a wider assessment in a specific territory. The combination of ANN models for emission estimation with geographical information tools could provide a significant knowledge advancement suitable to quantify, with higher accuracy, the impacts on the environment by identifying the areas with the highest risk and productions with the highest sustainability. This information could have high potential for decision-makers and policymakers to increase sustainability and reduce environmental impacts in the livestock sector. Despite progress in the application of ANNs within the livestock sector, certain gaps remain that need attention. Further research should prioritise the development of clearer and more detailed protocols for working with ANNs in livestock-related studies. Establishing comprehensive guidelines will enhance the consistency, reproducibility, and reliability of research outcomes, ultimately supporting the more effective adoption of ANN models in livestock management.

## Figures and Tables

**Table 1:** Contents of reviews.

Authors [Citation]	Year	Title	Time Period of the Articles Analysed	Number of Selected Articles	Aim or Focus	Review Methodology	Livestock Analysed	Type of Livestock Management	Gas Analysed	Instruments and Devices for Data Gathering	ML and ANN Models Applied	Main Results
Shine and Murphy [42]	2021	Over 20 years of Machine Learning Applications on Dairy Farms: A Comprehensive Mapping Study	1999–2021	129	Six research questions: (1) What countries/regions are responsible for the largest number of publications? (2) What journal and conference proceedings are research publications being published in? (3) What problem areas are being addressed using ML in the dairy farming domain? (4) What features are being applied to develop ML modes? (5) What ML algorithms are being utilised to develop the models? (6) Which evaluation metrics and methods are used?	Systematic mapping review	Dairy Cattle	Farm (Housing, Grazing, Pasture)	CH_4_	Undefined	ML: Bayes—Naive Bayes, Bayes net; Meta—Bagging, Adaboost; Rule—OneR; Statistical Regression—Logistic Regression, MLR, PLS, Linear Discriminant Analysis, Linear Regression, GAM; Tree—RF, Decision Tree, Gradient Boosting Machine, C4.5, CART, XGBoost; ANN: MLP, CNN, RNN	RQ3 Calving (Pregnancy, Conception Rate, Abortion, Reproduction Performance) Information = 23%; Cow Characteristics (Age, Weight, Breed, Genetics, Body Parameters, Medical Conditions) = 34%; Lactation Information = 19%; Milk Characteristics = 37%; Sensors = 48%; Soil Characteristics = 1%; Diet and Feeding = 11%; Milking Parameters = 10%; Meteorological Conditions = 14%; Other Variables = 7%; Farm Characteristics (Herd Size, Cooling System, Housing, Water Energy, Energy Balance, Ventilation) = 16%; RQ3: Physiology and Health = 27; Behaviour Analysis = 24; Accelerometer = 27; Image = 7; Pedometer = 6; RQ4: Tree-based Algorithms = 54%; ANN Algorithms = 50%; Statistical Regression-based Algorithms = 43%; Other types = 37%; Bayes Algorithms = 17%; Meta = 10%; Rule = 4%; Clustering = 1%; RQ6: RMSE = 56%; R2 = 46%; r = 27%; MAE = 24%; CCC = 17%; MAPE = 15%; MSE = 15%; RPE = 15%; MPE = 10%; MSPE = 7%
Rahman et al. [43]	2022	Prospect and scope of artificial neural network in livestock farming: a review	Undefined; From Table 1 emerged a timeframe from 2008 to 2022	Undefined; From Table 1 emerged a selection of 20 papers	Discover the potential implications of ANN in the different fields of animal science	Narrative review	Dairy Cattle, Beef Cattle, Buffalo, Sheep, Goat, Swine	Farm, Pasture	CH_4_, CO_2_, total gas emission	Undefined	CNN, RNN, Bayes NN, MLP	RQ3: ANN models can be applied in several livestock contexts, such as animal breeding, prediction of milk yield, evaluation of meat animals, inferring demography and recombination, genome-enabled prediction, animal nutrition, animal health and reproduction management, and in animal management in developing countries.
Bresolin and Dórea [46]	2020	Infrared Spectrometry as a High-Throughput Phenotyping Technology to Predict Complex Traits in Livestock Systems	Undefined; Authors specified that research ended in May 2020	113	Provide recent updates in MIR and NIR technologies and review analytical methods for spectral data analysis to improve predictive ability, different approaches to reduce data dimensionality, and impact of validation strategies on prediction	Systematic review	Dairy and Beef Cattle	Farm	CH_4_	Respiration chamber, sulphur hexafluoride tracer, and sniffer systems	PLS, Principal component regression, Bayes B, SVM, undefined ANN models	RQ6: R^2^ of ML methods (MLS, Bayes B) for CH_4_ emission ranged from 0.0 to 0.79
Jiang et al. [49]	2020	Analysis of Strategic Emission-Based Energy Policies of Developing and Developed Economies with Twin Prediction Model	1981–2012	23	Forecast CH_4_ emission and agricultural output (grown rate) by using Box–Jenkins and ANN methods and assess sustainability of CH_4_ emission vs. agricultural output	Systematic review	Undefined	Undefined	CH_4_, CO_2_, N_2_O, Green House Gases	Undefined	Statistical Box–Jenkins and Nonlinear autoregressive neural network (NAR) methods	RQ5: All applied methods have shown an increase in emission from non-OCSE countries (+30%), while OCSE countries are reducing their emission (−17.35%). Agricultural output trend is increasing for all countries (+62%)
Niloofar et al. [53]	2021	Data-driven decision support in livestock farming for improved animal health, welfare and greenhouse gas emissions: Overview and challenges	Undefined	Undefined	Provide an overview of the existing data-driven approaches in PLF and categorise them according to the different goals they aim for	Undefined	Cattle, Swine, Poultry	Farm	CH_4_, CO_2_, NH_3_, Green House Gases, N_2_O	Undefined	ML: KNN, SVM, Gaussian Mixture Models, Bayesian Network, RF; ANN: CNN, ANFIS, Undefined	RQ5: IPCC methodology lacks optimisation approaches to estimate GHG emission, but the ANNs’ estimation is accurate. Authors suggested applying several methods adapted to each farm to maximise the outputs

**Table 2:** Main features of the research studies analysed in this literature review.

No.	Authors	Years	Time Span	Study Area	Preprocessing	Neural Network	Validation Criteria	Training Algorithm	Software	Focus and Aims	Livestock/Source of Gas	Analysed Gas and Devices	Variables Analysed	Area of Research (Laboratory–Housing–Field)	Type of Gas Measurement/Most Significant Parameters	Type of ANN Approach	Results
1	Kolasa-Więcek [61]	2013	20 years	Poland	Undefined	Multilayer Perceptron	R^2^	Broyden–Fletcher–Goldfarb–Shanno	Statistica^®^—TIBCO Software	ANN model to assess N_2_O from direct soil emissions in relation to the use of crops and livestock population; Evaluate the most representative variables in the ANN model tested	Cattle; Horses; Poultry; Sheep; Swine; Goats	N_2_O obtained from undefined devices	(1) Input—arable land, permanent crop and pastures, livestock population output—direct N_2_O emissions (2) Input—wheat, barley, triticale, rye, maize, sugar beet, rapeseed, oats, potatoes, permanent meadows and pasture, livestock population × output—direct soil emissions	Open field	Emissions estimated in CO_2_ equivalent	Comparison of ANN hundreds of models: MLP 9–4–1 and MLP 16–5–1 reported as the best	MLP 16–5–1 = 98% Variables Sensitivity: Cattle 8.83, Swine 8.55, Rapeseed 7.05, Rye 6.09, Oats 4.95, Permanent meadow and pasture 4.65, Horses 4.06, Potatoes 3.53, Sheep 3.35, Mize 2.85, Wheat 2.46, Goats 2.38, Sugar beet 2.33, Poultry 2.21, Barley 1.95, Triticale 1.90
2	Martinez et al. [62]	2021	47 days	France	L2 Normalisation	Multilayer Perceptron	RMSE	Broyden–Fletcher–Goldfarb–Shanno	Python^®^	Evaluate sensitivity of Figaro^®^ resistances to CH_4_ versus the variables selected, combining low-cost devices with cross sensitivity variables to assess CH_4_ concentration and its variability	Manure	CH_4_ from tin-oxide Figaro^®^ resistances	CO_2_, water vapour, pressure, and temperature	Laboratory facility under controlled conditions	Concentrations expressed in ppm	Comparison of ANN models: only MLP 14–19 reported	MLP 14–19 = RMSE <0.2 Water vapour is the most important variable while CO have to be omitted to improve performance. However, Figaro^®^ resistances proved to be highly dependent for value of CO < 0.15 ppm and temperature < 26.5 °C
3	Lovanh et al. [63]	2014	1 month	USA	Variables selected with K-predictors in Statistica^®^—TIBCO Software	Multilayer Perceptron	MAE, MSE, SD	Broyden–Fletcher–Goldfarb–Shanno	Statistica^®^—TIBCO Software	Evaluate the effect of heat fluxes in NH_3_ emissions from waste lagoon	Swine	NH_3_ from INNOVA 1412 device	Surface temp, temp at 0.5 m, temp at 1.5 m, pH, moisture, pressure, wind speed and relative humidity	Farrowing pig farm	Emissions expressed in ppm	Comparison of ANN models: MLP 5–13–1, MLP 5–6–1, MLP 5–7–1, and MLP 5–15–1	MLP 5–7–1: MAE 0.018, MSE 0.001 and SD 0.031
4	Sun et al. [64]	2008	15 months	USA	PCA applied	Radial Basis Function Network	RMSE, MAE, R^2^	Undefined	MATLAB	Develop ANN model to predict air pollutant affected by the variables selected from piggeries	Swine	NH_3_ measured with chemiluminescence device, CO_2_ obtained from photoacoustic infrared analyser, PM_10_ obtained from tapered element oscillating microbalance, and H_2_S obtained from pulsed fluorescence sulphur device	Time of the day, season, ventilation rate, animal growth cycle, manure storage level, and weather conditions	Deep pit piggeries	Emissions expressed in Animal Unit and Concentrations expressed in ppm	Comparison of ANN models: Models were undefined	CO_2_ concentration model: R^2^ = 0.99; CO_2_ emission model: 0.93; H_2_S emission model: 0.92; NH_3_ concentration model: 0.91; NH_3_ emission, PM_10_ emission, and H_2_S concentration models between 0.88 and 0.81. MAE and RMSE undefined but reported as low by the authors
5	Lim et al. [65]	2007	3 years	Republic of Korea	PCA applied	Novel Piecewise-affine Network	MSE, R^2^	Backpropagation	MATLAB	Create a method to predict NH_3_ emissions and identify relative significance NH_3_ emissions factors	Field-applied manure	NH_3_ obtained from ALFAM database	Soil types, weather, manure characteristics, agronomic factors, and measuring techniques	Open field	Most important emissions factors: Wind speed, soil pH, average air temperature, and manure pH	Comparison of ANN (PWA-26) and statistical models (MLR)	PWA: Km = R^2^ 0.99; Nmax: R^2^ 0.99; MLR: Km = R^2^ 0.37; Nmax: R^2^ 0.66
6	Hempel et al. [66]	2019	2 years	Germany, Spain	Undefined	Multilayer Perceptron	R^2^	Backpropagation	Python^®^	Evaluate heat stress risks in dairy cattle applying ANN, ML and statistical models	Dairy Cattle	NH_3_ and CH_4_, obtained indirectly from environmental variables and heat stress	Temperature, relative humidity, zonal and meridional wind, sea level pressure, and global radiation	Dairy cattle farms	Emissions expressed in CO_2_ equivalent	Comparison of ANN (MLP up to 3 hidden layers; neurons were undefined), ML (RF, SVM) and statistical (LR) models	MLP: Dummerstorf = R^2^ 0.74; Groß Kreuz = R^2^ 0.56; Bétera = R^2^ 0.85; ML and statistical = undefined; Increasing of 2.9% (550 Gg) in Germany and 4.5% (353 Gg) in Spain
7	Hempel et al. [67]	2020	10 months	Germany	Undefined	Multilayer Perceptron	RMSE, MAE, TAE, R^2^	Backpropagation	Python^®^	ML models can best prediction NH_3_ emissions compared to statistical models; estimating the minimal temporal requirements for temporal sampling of training data; provide cons and pros of different ML approaches compared to ordinary statistical approaches	Dairy Cattle	NH_3_ obtained from Fourier Transform Infrared spectrometers	Hourly emission values derived from ventilation rate, time, temperature, wind speed and direction	Dairy cattle farms	Emissions expressed in Livestock Unit	Comparison of ANN (MLP: undefined), ML (SVM, XGBoost) and statistical (LR, RR) models	27 scenarios tested: best was 7th = MAE 0.480, RMSE 0.418, R^2^ 0.088; 13th = TAE 0.146
8	He et al. [68]	2023	21 years	China	Undefined	Backpropagation Neural Network	R^2^	Backpropagation	MATLAB	Accurate mathematical models to estimate emission from livestock excreta	Cattle, sheep, pigs, poultry, horses, donkey, mules, camels, and rabbit	Direct emission from fresh and dry excreta from undefined devices	Excreta rate, rearing cycle, moisture content, and commercial scale husbandry coefficient	Region of China	Emissions express in Unit (Mt/Year) of Dry and Fresh excreta	Comparison of ANN (undefined, Backpropagation Neural Network) and statistical (ARIMA) models	4 ANN model tested: Fresh manure = 0.93 RMSE; Dry Manure = 0.95 RMSE; Fresh manure from commercial-scale feedlot = 0.95 RMSE; Dry manure from commercial-scale feedlot = 0.98 RMSE; ARIMA: Fresh manure = 8.35 RMSE; Dry Manure = 7.20 RMSE; Fresh manure from commercial-scale feedlot = 7.30 RMSE; Dry manure from commercial-scale feedlot = 6.89 RMSE
9	Küçüktopcu and Cemek [69]	2021	NA	Turkey	Normalisation (Min–Max)	Multilayer Perceptron	SEP, RSR, AAPRE, R^2^, RMSE	Levenberg–Marquardt, Bayesian Regularisation, Scaled Conjugate Gradient	MATLAB	ANN model to assess CO_2_ emission, insulation thickness, and energy saving	Poultry	Mitigation of CO_2_ emissions from undefined devices	Annual total savings, heating degree days, optimum insulation thickness, reduction of CO_2_, total wall heat resistance, insulation materials, fuels, interest rate, and building lifetime	Poultry farm	Emissions expressed in Total CO_2_/Year	Comparison of ANN (MLP: 1 hidden layer with 8 to 15 neurons) and different training algorithms (LM, BR, and SCG)	3 ANN models (optimum insulation thickness, annual total net saving, and reduction of CO_2_ emission) evaluated for each training algorithms: OIT LM = 0.99 R^2^, 0.01 RMSE, 2.60 SEP, 0.03 RSR, 2.72 AAPRE;OIT BR = 0.99 R^2^, 0.01 RMSE, 3.70 SEP, 0.05 RSR, 3.44 AAPRE; OIT SCG = 0.99 R^2^, 0.01 RMSE, 4.22 SEP, 0.06 RSR, 10.87 AAPRE; ATS LM = 0.99 R^2^, 0.94 RMSE, 5.58 SEP, 0.04 RSR, 8.18 AAPRE;ATS BR = 0.99 R^2^, 1.70 RMSE, 10.15 SEP, 0.07 RSR, 10.47 AAPRE; ATS SCG = 0.99 R^2^, 1.97 RMSE, 11.79 SEP, 0.89 RSR, 14.34 AAPRE; RCO2 LM = 0.99 R^2^, 1.04 RMSE, 1.72 SEP, 0.05 RSR, 4.17 AAPRE;RCO_2_ BR = 0.99 R^2^, 1.62 RMSE, 2.67 SEP, 0.08 RSR, 6.47 AAPRE; RCO2 SCG = 0.99 R^2^,1.98 RMSE,3.26 SEP, 0.09 RSR, 10.86 AAPRE
10	Basak et al. [70]	2022	3 months	Republic of Korea	Z-score Normalisation	Backpropagation Network	RMSE, R^2^	Backpropagation	Python^®^	Modelling CH_4_ manure emission using statistical and machine learning methods	Swine	CH4 obtained from IPCC tier 2 equation approach	Mass of pigs, age, and feed intake	Piggeries	Emissions expressed in Pig/kg×*Year	Comparison of ANN (BPNN: undefined), ML (RF) and statistical (MLR, PL, RR) models	Best value of models: MLR = R^2^ 0.90, RMSE 0.01; PR = R^2^ 0.91, RMSE 0.01; RR = R^2^ 0.92, RMSE 0.01; RF = R^2^ 0.97, RMSE 0.01; ANN = R^2^ 0.90, RMSE 0.01
11	Park et al. [71]	2023	10 months	Republic of Korea	Sliding window	Recurrent Neural Network, Convolutional Neural Network, Transformer Neural Network	MAE	Undefined	NA	Comparative analysis of ANN models to predict NH_3_ concentrations	Swine	NH_3_ measured with INNOVA 1512i	Ventilation rate, temperature, RH, and NH_3_	Gestation pig facilities	Concentrations expressed in ppm	Comparison of ANN (MLP = undefined; RNN = 3 hidden layers with 64 neurons; CNN = 1D; Transformer = undefined) models	Input = 1 week, output = 1 week: (MLP = 2.15 MAE, RNN = 1.83 MAE, CNN = 2.02 MAE, Transformer = 1.89 MAE); input = 1 week, output = 2 week: (MLP = 2.24 MAE, RNN = 1.78 MAE, CNN = 1.92 MAE, Transformer = 1.90 MAE); input = 1 week, output = 3 week: (MLP = 2.20 MAE, RNN = 1.95 MAE, CNN = 1.89 MAE, Transformer = 1.87 MAE); input = 1 week, output = 4 week: (MLP = 2.15 MAE, RNN = 1.79 MAE, CNN = 1.87 MAE, Transformer = 1.73 MAE)
12	Genedy et al. [72]	2023	3 years and 2 months	USA, Switzerland	Undefined	Recurrent Neural Network	RMSE, MAE	Undefined	Python^®^	Modelling ANN structure to assess NH_3_ from manure storage	Dairy Cattle	NH_3_ obtained from tuneable diode laser spectrometer	Animal numbers, air temperature, wind speed and direction, manure temperature, and pH	Dairy cattle farms	Emissions expressed in g×m2/d	Comparison of ANN (undefined—PI–LSTM, HT–CPBM, and Base–CPBM) models	2 datasets applied—flushed lagoon (Base–CPBM = 2.19 MAE, 3.34 RMSE, HT–CPBM = MAE 1.40, RMSE 2.38, and PI–LSTM = 1.23 MAE, 2.20 RSME); steel tank (Base–CPBM = 1.29 MAE, 2.42 RMSE, HT–CPBM = MAE 1.26, RMSE 2.39, and PI–LSTM = 0.97 MAE, 1.64 RSME)
13	Chen et al. [60]	2022	26 years	UK	Normalisation (Min–Max)	Feedforward Network	R^2^, RMSE, Concordance Correlation Coefficient	Backpropagation	R	Comparing statistical and ML models to predict nitrogen excretion from manure	Dairy Cattle	Nitrogen excretion	Nitrogen intake, dietary Nitrogen intake, milk yield, dietary forage proportion, live weight, and diet metabolizable energy content	Dairy cattle farms	Nitrogen excretion	Comparison of ANN (FNN: from 1 to 3 hidden layer, and from 1 to 6), ML (RF, SVM) and statistical (MLR) models	MLR = 44.7 RMSE, 0.60 CCC; RF = 46.8 RMSE, CCC 0.58; SVM = 44.9 RMSE, CCC 45.3; ANN 34.7 RMSE, CCC 0.70
14	Besteiro et al. [73]	2017	78 days	Spain	Bayesian Information Criterion	Feedforward Network	RMSE	Backpropagation	R	Modelling ANN structure to assess CO_2_ from piglet facilities	Swine	CO_2_ obtained from Delta Ohm HD37BTV.1 transmitter	CO_2_ concentration in animal zone, Variation of CO_2_ concentration in animal zone, and external temperature	Piggeries	Concentrations expressed in ppm	Comparison of ANN (undefined) models	ANN = 26.33 RMSE, 1.26% MARE, 0.99 r, and 0.99 IA
15	Shi et al. [74]	2024	1 day	China	Normalisation (Min–Max)	Recurrent Neural Network, Backpropagation Neural Network, Particle Swarm-Optimised Backpropagation Neural Network	R^2^, MAE, RMSE	Backpropagation	NA	Combining electric nose in bionic chamber with ANN model to detect NH_3_ emissions	Livestock excreta	NH_3_ and Ethanol obtained from SMD1002 and SMD1005 sensors installed in National Instrument USB6289 (Emerson Electric Co., St. Louis, USA) with 10 Hz frequency	Different concentrations of NH_3_ and Ethanol, collected with different sensors	Laboratory facility under controlled conditions	Emissions expressed in ppm	Comparison of ANN (undefined—RNN, BPNN, and PSO-BPNN) models	All ANN structures were undefined—BP = NH_3_ 0.99 R^2^, 5.67 MAE, 8.10 RMSE; Ethanol 0.99 R^2^, 2.38 MAE, 3.04 RMSE; RNN = NH_3_ 0.96 R^2^, 9.47 MAE, 17,97 RMSE; Ethanol 0.99 R^2^, 4.78 MAE, 6.04 RMSE; PSO-BP = NH_3_ 0.96, 0.23 MAE, 16.67 RMSE; Ethanol 0.99 R^2^, 3.56 MAE, 5.33 RMSE
16	Stamenković et al. [75]	2015	8 years	20 European countries ^1^	Undefined	General Regression Neural Network, Backpropagation Network	MAE, RMSE, Index of Agreement, Pearson Correlation Coefficient	Backpropagation	NA for ANN; IBM SPSS Statistic for Windows for MLR	Modelling ANN to estimate CH_4_ emission	Cattle	CH_4_ obtained from EDGAR database	Gross domestic product, waste deposit, municipal waste generation, land use, number of cattle, primary production of gas, and CH_4_ emissions	Country based	Emissions expressed in kg per capita	Comparison of ANN (undefined—BPNN, GRNN) and statistical (MLR) models	BPNN = 1.00 IA, 3.4 MAE, 5.0 RMSE, 0.97 r; GRNN = 0.97 IA, 3.6 MAE, 7.0 RMSE, 0.94 r; MLR = 0.83 IA, 11.3 MAE, 14 RMSE, 0.75 r
17	Shadpour et al. [76]	2022	5 years	Canada, Denmark	Normalisation (Min–Max)	Multilayer Perceptron (LMANN, BRANN, SCGANN)	RMSE, Pearson Correlation Coefficient, Residual Prediction Deviation	Levenberg–Marquardt, Bayesian Regularisation, Scaled Conjugate Gradient	MATLAB	Predicting CH_4_ emission from common device with ANN models	Dairy Cattle	CH_4_ obtained from Mid-Infrared Reflectance Spectroscopy	Age at calving, milk yield, fat yield, protein yield, and mid-infrared spectroscopy	Dairy cattle farms	Emissions expressed as weekly average	Comparison of ANN (LMANN, BRANN, SCGANN all with 1 hidden layer) and statistical (PLS, models	PLS = 0.255 PCC, 90.45 RMSE, 1.21 RPD; LMANN = 0.360 PCC, 93.32 RMSE, 1.10 RPD; BRANN = 0.320 PCC, 95.21 RMSE, 1.08 RPD; SCGANN = 0.330 PCC, 97.20 RMSE, 1.06 RPD
18	Peng et al. [77]	2022	1 month	China	Normalisation (Min–Max)	Recurrent Neural Network, Backpropagation Neural Network	MAE, RMSE, R^2^	Backpropagation	Python^®^	PredictingNH3 from piggeries applying ANN and ML approaches	Swine	NH_3_ obtained from INNOVA 1412i	NH_3_, CO_2_, H_2_O, pressure, outdoor temperature, indoor ventilation, indoor temperature, indoor humidity, and outdoor rainfall	Piggeries	Concentrations expressed in ppm	Comparison of ANN (undefined—BPNN, RNN, PSO-BPNN, PSO-RNN) and ML (SVM, XGBoost) models	RNN = 0.92 R^2^; BPNN = 0.80 R^2^; SVM = 0.89 R^2^; XGBoost = 0.92 R^2^; PSO-RNN = 0.96 R^2^, 0.61 RMSE

^1^ = Bulgaria, Czech Republic, Denmark, Estonia, Greece, Spain, France, Latvia, Lithuania, Luxembourg, Hungary, The Netherlands, Austria, Poland, Portugal, Romania, Slovenia, Slovakia, Finland, UK; NA = not available; R^2^ = correlation coefficient; TAE = total absolute error; MAE = mean absolute error; RMSE = root mean square error; MSE = mean square error; SD = standard deviation; SEP = standard error of prediction; RSR = observations standard deviation ratio; AAPRE = average absolute percent relative error, PCC = Pearson correlation coefficient; RPD = residual prediction deviation; IA = index of agreement; CCC = concordance correlation coefficient; MARE = mean absolute relative error.

**Table 3:** Data for concentration measurements and emission estimation methodology. The acronym N.A. indicates that the information was not available in the manuscripts analysed.

Articles	Emission		Concentrations					
	Estimation Method	Tracer Gas	Measurement Methodologies	Measurement Duration	Devices	Frequency of Measurement	Calibration	Measurement Location
Kolasa-Więcek [61]	CO_2_ equivalent	N.A.	Data Obtained from FAO, Ifa, and UNFCCC Databases		N.A.	N.A.	N.A.	N.A.
Martinez et al. [62]	N.A.	N.A.	Gas sensing through voltage measuring	47 days	Figaro TGS (2600, 2611-C00, 2611-E00), Sensirion SHT75, Bosch BMP180	5 min	Yes	N.A.
Lovanh et al. [63]	Mass balance	N.A.	Photoacoustic Gas Analyzer	1 month	Innova 1412, HOBO weather station,	70 s	Yes	0.5 m above lagoon
Sun et al. [64]	Mass balance	N.A.	Chemiluminescence, Photoacoustic Infrared, Tapered Element Oscillating Microbalance	N.A.	Model 17C Thermal Environment Instruments, Model 45C Thermal Environment Instruments	N.A.	N.A.	N.A.
Lim et al. [65]	Mass balance	N.A.	Data Obtained from Database (ALFAM, DIAS, IMAG, IGER, ADAS, CRPA)	N.A.	N.A.	N.A.	N.A.	N.A.
Hempel et al. [66]	CO_2_ equivalent	N.A.	Environmental Data from Database (DWD, NCDC, NOAA)	N.A.	N.A.	N.A.	Yes	3, 4, 6 m from floor
Hempel et al. [67]	Mass balance	N.A.	Infrared Spectrometry	10 months	Gasmet (CX4000)	10 min	Yes	3.2, 4, 6 m from floor
He et al. [68]	Mass balance	N.A.	Data Obtained from National Database (China’s Statistical Yearbooks)	N.A.	N.A.	N.A.	N.A.	N.A.
Küçüktopcu and Cemek [69]	Mass balance	N.A.	Data Obtained from Database	N.A.	N.A.	N.A.	N.A.	N.A.
Basak et al. [70]	Mass balance	N.A.	Weather Sensing, pH Meter, Electronic Mass Balance	3 months	MetPRO, HP9010, FX-300iWP	24 h	N.A.	N.A.
Park et al. [71]	N.A.	N.A.	Photoacoustic Gas Analyzer, Ventilation Measuring Device, Environmental Parameters	N.A.	Innova 1512i, VelociCalc Air Velocity Meter 9535, Undefined indoor sensors	N.A.	Yes	N.A.
Genedy et al. [72]	Mass balance	N.A.	Spectrometry Laser	3 years and 2 months	GasFinder2	10 min	N.A.	1 m below, 1, 2, 3 m above tank storage
Chen et al. [60]	N.A.	N.A.	Data Obtained from Previous Studies	N.A.	N.A.	N.A.	N.A.	N.A.
Besteiro et al. [73]	N.A.	N.A.	Weather Sensing, Gas Sensing	78 days	HOBO, Delta Ohm HD37BTV.1	10 min	N.A.	0.2 m above separation slats
Shi et al. [74]	Mass balance	N.A.	Experimental Electronic Nose	1 day	USB6289, LZB-4WB, SMD 1002, SMD 1005	3 min	Yes	N.A.
Stamenković et al. [75]	Mass balance	N.A.	Data Obtained from Annual National Database	N.A.	N.A.	N.A.	N.A.	N.A.
Shadpour et al. [76]	Mass balance	N.A.	Infrared Spectrometry, Sniffer	N.A.	MilkoScan FT+	N.A.	Yes	N.A.
Peng et al. [77]	N.A.	N.A.	Photoacoustic Gas Analyzer, Environmental Parameters	1 month	Innova 1412i, HOBO	3 min (gas), 5 min (environment)	N.A.	1.7 m from floor

**Table 4:** Information on livestock, building, and management. The acronym N.A. indicates that the information was not available in the manuscripts analysed.

Articles	Species	Breed	Number of Animals	Housing System	Ventilation System	Feed Composition	Manure Parameters	Manure Management
Kolasa-Więcek [61]	Cattle, Horses, Poultry, Sheep, Swine, Goats	N.A.	N.A.	N.A.	N.A.	N.A.	N.A.	N.A.
Martinez et al. [62]	N.A.	N.A.	N.A.	N.A.	N.A.	N.A.	N.A.	N.A.
Lovanh et al. [63]	Swine	N.A.	2.000	Indoor Farrowing Piggeries	N.A.	N.A.	N.A.	Anaerobic Waste Lagoon
Sun et al. [64]	Swine	N.A.	960	Indoor Fattening Piggeries	Force Ventilated	N.A.	N.A.	N.A.
Lim et al. [65]	N.A.	N.A.	N.A.	N.A.	N.A.	N.A.	Dry matter, Total Ammoniacal Nitrogen, pH	N.A.
Hempel et al. [66]	Cattle	Holstein-Friesian	606	Indoor Cattle Barn	Naturally Ventilated	N.A.	N.A.	N.A.
Hempel et al. [67]	Cattle	Holstein-Friesian	355	Indoor Loose Cattle Barn	Naturally Ventilated	Soy, Oilseed Rape, Maize, Rye, Lupins	N.A.	N.A.
He et al. [68]	Cattle, Sheep, Swine, Poultry, Horses, Donkey, Mules, Camels, Rabbit	N.A.	N.A.	N.A.	N.A.	N.A.	Excreta Rate, Moisture	N.A.
Küçüktopcu and Cemek [69]	Poultry	N.A.	N.A.	N.A.	N.A.	N.A.	N.A.	N.A.
Basak et al. [70]	Swine	Yorkshire	18	Indoor Piglet Barn	Force Ventilated	Crude Protein, Crude Fat, Crude Fibre, Crude Ash, Calcium, Phosphorus, Lysine, Digestible Crude Protein, Digestible Energy	pH, Moisture, Dry Matter, Ash, Volatile Solid Daily Excretion Rate	Roof Manure Collector
Park et al. [71]	Swine	N.A.	464	Indoor Farrowing Piggeries	Force Ventilated	Crude Protein, Crude Fat, Crude Fibre, Crude Ash, Calcium, Phosphorus, Lysine	N.A.	Roof Manure Collector
Genedy et al. [72]	Cattle	N.A.	2.680	Indoor Free-Stall Cattle Barn	Naturally Ventilated	N.A.	Temperature, pH	Waste Lagoon, Steel Tank
Chen et al. [60]	Cattle	Holstein-Friesian, Holstein crossbreed, Norwegian and Swedish Red	951	Indoor Free-Stall Cattle Barn	N.A.	Grass Silage, Fresh Grass, Maize Silage, Whole Crop Wheat Silage	N.A.	N.A.
Besteiro et al. [73]	Swine	Large White x Landrace	800	Indoor Piglet Barn	Force Ventilated	N.A.	N.A.	N.A.
Shi et al. [74]	N.A.	N.A.	N.A.	N.A.	N.A.	N.A.	N.A.	N.A.
Stamenković et al. [75]	Cattle	N.A.	N.A.	N.A.	N.A.	N.A.	N.A.	N.A.
Shadpour et al. [76]	Cattle	Holstein-Friesian	202	Indoor Cattle Barn	N.A.	N.A.	N.A.	N.A.
Peng et al. [77]	Swine	N.A.	220	Indoor Fattening Piggeries	Force Ventilated	N.A.	N.A.	N.A.

**Table 5:** Validation criteria and range of applications.

Validation Criteria	N. of Papers
AAPRE	1
CCC	1
IA	1
MAE	7
MSE	2
PCC	1
R^2^	10
RMSE	12
RPD	1
RSR	1
SD	1
SEP	1
TAE	1
MARE	1

## Data Availability

No new data were created or analyzed in this study. Data sharing is not applicable to this article.

## Abbreviations

The following abbreviations are used in this manuscript:

NH_3_	Ammonia
ANN	Artificial Neural Network
PLF	Precision Livestock Farming
MLP	Multilayer Perceptron
CO_2_	Carbon Dioxide
CH_4_	Methane
